# CScape: a tool for predicting oncogenic single-point mutations in the cancer genome

**DOI:** 10.1038/s41598-017-11746-4

**Published:** 2017-09-14

**Authors:** Mark F. Rogers, Hashem A. Shihab, Tom R. Gaunt, Colin Campbell

**Affiliations:** 1 Intelligent Systems Laboratory, University of Bristol, Bristol, BS8 1UB United Kingdom; 2 MRC Integrative Epidemiology Unit (IEU), University of Bristol, Bristol, BS8 2BN United Kingdom

## Abstract

For somatic point mutations in coding and non-coding regions of the genome, we propose *CScape*, an integrative classifier for predicting the likelihood that mutations are cancer drivers. Tested on somatic mutations, *CScape* tends to outperform alternative methods, reaching 91% balanced accuracy in coding regions and 70% in non-coding regions, while even higher accuracy may be achieved using thresholds to isolate high-confidence predictions. Positive predictions tend to cluster in genomic regions, so we apply a statistical approach to isolate coding and non-coding regions of the cancer genome that appear enriched for high-confidence predicted disease-drivers. Predictions and software are available at http://CScape.biocompute.org.uk/.

## Introduction

Next generation sequencing technologies have accelerated the discovery of single nucleotide variants (SNVs) in the human genome, stimulating the development of predictors for classifying which of these variants are likely functional in disease, and which neutral. Predictors have been developed for variants in both the coding and non-coding regions of the human genome. For example, in Shihab *et al*.^1^, we developed such a predictor based on pathogenic disease-driver germline variants from the Human Gene Mutation Database (HGMD^2^), and assumed neutral variants from the 1,000 Genomes Project Consortium (1000G^3^). Multiple types of data may be informative, so we used an integrative binary classifier that weighted component data-types according to their relative informativeness^1^. A variety of similar predictors have been proposed^4–9^.

In this paper we focus on prediction for somatic point mutations in both coding and non-coding regions of the human cancer genome. The development of such predictors will be important for interpreting cancer sequence databases currently being compiled, such as the Cancer Genome Atlas^10^, the International Cancer Genome Consortium^11^ and national programmes such as the Genomics England (100,000 genomes) Project^12^. Mirroring previous methods^1, 13^, we use an integrative classifier and a wide variety of data sources. Using leave-one-chromosome-out cross-validation (LOCO-CV), the proposed method, which we call *CScape*, outperforms rival methods, achieving balanced accuracy of 72.3% in coding regions and 62.9% in non-coding regions. Tested on independent data sets, *CScape* can achieve up to 91% balanced accuracy in coding regions and 70% in non-coding regions. Most methods in this domain attempt to rank mutations to identify the most likely oncogenic examples. One drawback of this approach is that clinical practitioners may find it difficult to interpret scores without provision of specific thresholds for discriminating between benign and oncogenic mutations. Hence in this study we establish two thresholds for each model (a default threshold and a high-confidence threshold) by associating a confidence measure to each predicted label. If we restrict prediction to highest confidence instances only (*cautious classification*) then balanced accuracy in LOCO-CV rises to 91.7% for coding regions and 76.1% for non-coding regions, with predictions confined to 17.7% (coding) and 14.8% (non-coding) of nucleotide positions across the genome, respectively. These high confidence positive (oncogenic) predictions are typically clustered by genomic location, hence we further introduce a statistical method to find significant contiguous sets of such positive predictions. This latter approach highlights a number of genes as potentially oncogenic via somatic point mutation.

## Results

We assembled two distinct datasets: our pathogenic (positive) dataset was constructed using somatic point mutations from the COSMIC database^14^ (version 75, November 2015) and our control (negative) dataset was constructed using SNVs from the 1,000 Genomes Project^3^. This control dataset contains many variants which are unannotated and therefore could be true positives. Similarly, we may expect true negatives among the positively labeled COSMIC datapoints, such as passenger mutations co-located with driver mutations. Among the COSMIC database annotations is the recurrence level, or the number of times a mutation has been observed in different cases. To increase the likelihood that positive examples are drivers, not passengers, we use pathogenic examples with a COSMIC recurrence count as high as possible. However, the number of training examples decreases rapidly as we increase the recurrence threshold (Supplementary Figure S1). In addition, an exceedingly high threshold may introduce bias, for example, by restricting the training set to only a subset of relevant genes. Hence for each model we choose as our threshold the highest recurrence level that shows little evidence of bias and provides sufficient training examples to create an accurate classifier (Supplementary Figure S2). Using this approach we thus select recurrence thresholds of *r* = 5 for coding regions, and *r* = 3 for non-coding regions.

Bias may also be introduced if negative examples are located in different genomic regions from positive examples. For example, if positives appear predominantly near transcription start sites while negatives are distributed more broadly^1, 7, 15^. To ensure the locations of putative non-driver mutations approximate those of driver mutations, we select only those putative non-drivers found within a window *w* of a putative driver mutation. For coding examples we use *w* = 10,000, and for non-coding examples we use *w* = 1,000 (Supplementary Figure S3). Hence our final training set, outlined in Supplementary Table S1, consists of 46,420 coding examples and 131,714 non-coding examples.

### Feature Groups

All of our data are based on the GRCh37/hg19 version of the human genome and detailed further in the Supplementary. Following our previous work^1, 13^, we annotated our datasets using more than 30 *feature groups* that could be predictive of pathogenicity (a detailed description of these feature groups can be found in the Supplementary):

- *Genomic*: genomic features included GC content, sequence spectra^16^, repeat regions and measures of region uniqueness.
- *Evolutionary*: as in previous work, evolutionary features included a comprehensive set of conservation-based measures provided by tools such as Phastcons^17^, PhyloP^18^ and FATHMM^1^.
- *Consequences (coding only)*: using the Variant Effect Predictor^19^ we used binary vectors to represent allele consequences and the affected amino acids within all transcripts associated with a mutation.

In addition to these feature groups, we developed new features specifically to boost performance for our non-coding predictor, though similar features proved effective in both coding and non-coding regions (see Supplementary Methods for a complete description). Briefly, these new features fall into two categories: *sequence* features designed to capture disruption that occurs in a sequence as a consequence of a mutation, and *genomic context* features that encapsulate genomic features within a mutation’s neighborhood, such as known splice sites or start/stop codons. Recent studies have identified regulatory elements, such as transcription factor binding sites (TFBS), as potential targets of oncogenic mutations in non-coding regions^20^. Consequently at least one method, FunSeq2, already incorporates motif-specific analysis to score mutations in non-coding regions^21^. The COSMIC database also provides a set of *mutational signatures* that are specific to oncogenic mutations. These are associated with various distinct forms of mutation, such as DNA replication errors, defective DNA repair, enzymatic DNA modification, and exposure to mutagens^22^. However, this signature set is relatively new and hence may represent only a subset of potential oncogenic driver signals. Further, the metrics used to develop these signatures are based in part on the COSMIC database and potentially could introduce bias into our models.

### CScape models

We evaluated all models using LOCO-CV, omitting mitochondrial and allosomal (X and Y) chromosomes from testing as these tended to yield few examples, and we wanted to focus on performance within autosomal chromosomes. For each fold we leave out one test chromosome while the remaining 21 chromosomes are used to train the model, using the same model parameters for all folds. Except where noted, we trained models using randomly selected, balanced sets of 1,000 positive and 1,000 negative examples. This relatively small subset of examples yields accuracy nearly as high as with complete training sets but takes less time to train, and allows us to estimate the variability of the test results. For testing we used all available examples for the left-out chromosome, resulting in unbalanced test sets for coding but nearly balanced test sets for non-coding (Supplementary Table S1). For this reason we report balanced accuracy for all tests.

We integrated data from the feature groups outlined above and used them to train two distinct classifiers: *CS-coding* for coding regions, and *CS-noncoding* for noncoding regions. The simplest kernel method for integrating different data sources is to combine features from all sources into a single kernel. This allows a model to learn how features from one source may interact with features from other sources. Given at least 30 possible data sources, the number of possible combinations of feature groups makes exhaustive testing impractical. Instead, we use an approach similar to previous work in which we found that greedy sequential learning could be an effective means to identify an optimal combination of groups^13^. To identify the data sources to include in each model, we first build a model based on two top-ranked data sources combined into a single kernel and record its balanced test accuracy in LOCO-CV. We build subsequent models by adding data sources in descending order of their individual balanced accuracy in LOCO-CV. We select as our final combination those data sources associated with a peak or a plateau in LOCO-CV balanced accuracy (Supplementary Figure S6). We evaluate all combinations with and without data normalisation, where we standardise features by subtracting the mean and dividing by the standard deviation. For these models we observed no difference in performance between the raw feature values and standardised data. The final *CS-noncoding* model includes just five feature groups: *46-way conservation*, *100-way conservation*, *Spectrum*, *Genomic context* and *Mappability* (Supplementary Table S4, Supplementary Figure S6). For *CS-coding* the best model also included five feature groups, but a slightly different set: *VEP* (including amino acid substitutions), *46-way conservation*, *100-way conservation*, *Genomic context* and *Spectrum*.

In previous studies we found that integration at the kernel level, using multiple kernel learning (MKL)^23, 24^ and sequential learning strategies, tended to outperform integration at the data level^13, 25^. We thus applied our sequential learning pipeline^13^ to examples in both coding and non-coding regions: we ranked feature groups by balanced validation accuracy and added them incrementally to aggregate kernels. For non-coding examples, the best MKL aggregates achieved just over 58% balanced accuracy (Supplementary Figure S7, left). For coding examples, the best achieved over 65% balanced accuracy (Supplementary Figure S7, right). However, in both cases, data-level integration yielded greater balanced accuracy than kernel-level integration.

**Figure 1:**
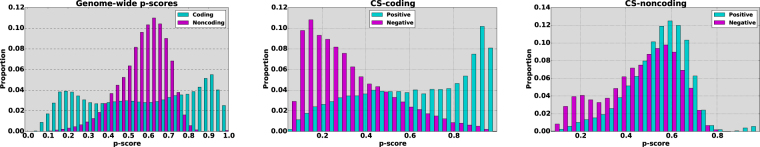
Distributions of CScape p-scores reveal distinct differences in the behaviours of the *CS-coding* and *CS-noncoding* models. **Left:** *CS-coding* tends to make more confident predictions, as its *p*-scores exhibit a bi-modal distribution with peaks at 0.18 and 0.91. By contrast *CS-noncoding* makes few confident predictions, attesting to the difficulty in discriminating between oncogenic and benign mutations in these regions. **Middle:** *p*-score distributions for positive and negative examples in the *CS-coding* LOCO test set are substantially different, further demonstrating that the classes are separable to some degree. **Right:** *p*-score distributions for *CS-noncoding* are highly similar; despite careful selection of informative feature groups, clear differences between the classes remain elusive.

**Figure 2:**
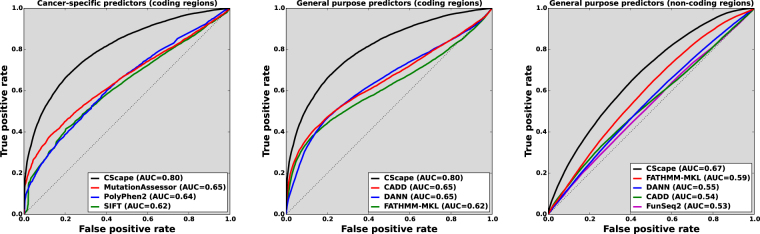
ROC curves for COSMIC data show *CScape’s* performance relative to other state-of-the-art models in coding regions (left & middle) and non-coding regions (right). **Left:** *CS-coding* compares favourably with other methods specifically designed to predict SNVs in the coding regions of the cancer genome. **Middle:** *CS-coding* also exhibits better performance than popular general-purpose classifiers when tested on putative oncogenic examples. **Right:** In non-coding regions we also see performance gains using *CS-noncoding*, here compared with three general purpose predictors, FATHMM-MKL^1^, CADD^7^, DANN^8^ and cancer-specific FunSeq2^21^.

Both of our models output probability scores on [0, 1]: scores below 0.5 are predicted as negative and scores above 0.5, positive (see Methods). These scores (herein called *p-scores*) lead to a natural interpretation, such as identifying *p*-scores above 0.95 as high-confidence predictions. The two models exhibit markedly different *p*-score distributions across the genome (Fig. 1, left): *CS-coding* yields *p*-scores in a bi-modal distribution with peaks at 0.18 and 0.91, demonstrating that a substantial portion of its predictions will be highly confident. By contrast, the *CS-noncoding* model makes few confident predictions, with a majority of *p*-scores clustered close to 0.6. When we examine the relative distributions of positive and negative examples from the LOCO test sets, the differences between the coding and non-coding models becomes clearer. *CS-coding p*-score distributions are markedly different between positive and negative examples, exhibiting some separation between the classes (Fig. 1, middle). By contrast, *CS-noncoding p*-score distributions are highly similar (Fig. 1, right). We have identified feature groups that enable the non-coding model to discriminate between classes with greater balanced accuracy than previous methods (Fig. 2), but achieving clear separation remains challenging. We note that a small proportion of non-coding predictions carry high confidence. Multiplied by the large number of non-coding positions in the human genome, this translates into a large population of high-confidence predictions in non-coding regions.

Applying LOCO-CV to the COSMIC training data, we obtained the performance presented in Table 1 and Fig. 2. Performance at the default threshold is good relative to alternative methods, though overall balanced accuracy (62% for non-coding mutations and 73% for coding mutations) leaves room for improvement, also reflected in the corresponding Matthews correlation coefficients (MCC) of 0.25 and 0.46, respectively. Cautious classification (see below) improves these measurements substantially, raising balanced accuracy by 14 percentage points for the non-coding model and by 19 percentage points for the coding model, and doubling (non-coding) or nearly doubling (coding) the MCC.

**Table 1:** Statistics for *CS-noncoding* and *CS-coding* applied to LOCO-CV test data provide estimates of how the models are likely to perform on new examples.

Classifier	TP	FP	TN	FN	Sens.	Spec.	Bal. Acc.	MCC	PPV
*CS-noncoding*	48,079	30,902	36,363	20,432	0.70	0.54	0.62	0.25	0.61
cautious (*τ* = 0.70)	6,512	1,859	13,044	3,544	0.65	0.88	0.76	0.54	0.78
*CS-coding*	10,333	3,992	13,297	4,631	0.69	0.77	0.73	0.46	0.72
cautious (*τ* = 0.89)	3,398	197	1,319	124	0.97	0.87	0.92	0.85	0.95

Shown are the true positive (**TP**), false positive (**FP**), true negative (**TN**) and false negative (**FN**) counts for the two classifiers. Also shown are performance statistics: sensitivity (**Sens**., the proportion of positive examples correctly classified), specificity (**Spec**., the proportion of negative examples correctly classified), balanced accuracy (**Bal. Acc**.), the Matthews correlation coefficient (**MCC**) and the positive predictive value (**PPV**, the number of true positive predictions over the number of positive predictions). *τ* is the cutoff on the confidence for cautious classification.

### Comparison with other Methods

Cancer specific predictors have been proposed for prediction in coding regions of the cancer genome^4, 5, 26^. General purpose predictors have also been proposed for prediction across the entire genome (coding and non-coding regions) using catalogued disease-drivers across a variety of disease traits (e.g. HGMD^2^). However, there is currently a dearth of predictors specifically trained for the non-coding regions of the cancer genome. In Fig. 2 we present ROC curves demonstrating that *CS-coding* and *CS-noncoding* both outperform rival prediction tools on the COSMIC data. Figure 2 (left) shows *CS-coding* compared with cancer-specific classifiers (e.g., PolyPhen2^4^ and SIFT^5^ scores adjusted for somatic mutations via TransFIC^27^). In Fig. 2 (middle) we compare *CS-coding* with well established general-purpose pathogenic mutation classifiers (e.g., CADD^7^, DANN^8^). In Fig. 2 (right) we compare *CS-noncoding* with both general-purpose classifiers (FATHMM-MKL^1^, CADD^7^, DANN^8^) and the cancer-specific method FunSeq2^21^. With an area-under-curve measure (AUC) of 0.67 against its nearest competitor with 0.59 (our FATHMM-MKL^1, 9^), *CS-noncoding* markedly outperforms these existing methods. The *CScape* tool at the accompanying website incorporates both *CS-coding* and *CS-noncoding*.

### Cautious Classification

**Figure 3:**
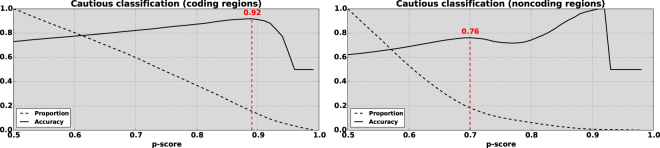
Cautious classification curves are used to select thresholds that maximise each model’s balanced accuracy. Solid curves show balanced test accuracy (*y*-axis) as a function of the *p*-score cutoff (confidence, *x*-axis). Dashed curves show the proportion of examples in the *test* datasets where such predictions could be made. **Left:** For *CS-coding*, the peak test accuracy is 91.7%, at a *p*-score of 0.89, with predictions for 15.6% of examples. **Right:** For *non-coding* prediction (intergenic and intragenic) the peak test accuracy is 76.1% at a *p*-score of 0.70, with 18.4% of examples predicted. Beyond the peak, the curve is of decreased reliability since the test set becomes very small.

By associating a confidence measure with each prediction, we can evaluate balanced test accuracy against possible confidence measure cutoffs. For each cutoff threshold *τ* ∈ [0.5, 1] we accept a subset of predictions where *p* ≥ *τ* are positive (drivers) and *p* ≤ (1 − *τ*) are negative (passengers). We compare these refined sets of predictions to other models in the Supplementary. Figure 3 shows balanced accuracy as a function of *p*-score cutoff for the coding (*left*) and non-coding (*right*) predictors in LOCO-CV, along with the proportion of examples whose *p*-scores exceed the cutoff. For *CS-coding* (left), the peak balanced test accuracy is 91.74% which is achieved for a cutoff on the confidence measure at *τ* = 0.89. With the data used in cross-validation, 15.62% of test examples (5,038 out of 32,253) had a high enough confidence for prediction. Taken across the entire genome, 17.7% of locations in coding regions have a predicted label at this level of confidence. For *CS-noncoding* (right) the curve has two peaks, as balanced accuracy declines for thresholds between *τ* = 0.70 and *τ* = 0.81. In this region small sample size effects begin to influence evaluation of the balanced test accuracy. We choose the lower of the two peaks for the threshold as higher *p*-scores are of decreasing reliability. At *τ* = 0.70 the balanced test accuracy is 76.14% for 18.38% of test examples (24,209 out of 131,714). Taken across the entire genome 14.8% of *p*-scores in non-coding locations had values greater than 0.70.

### Evaluation on previously unseen non-coding examples

To evaluate our models on previously unseen examples in non-coding regions, we downloaded putative somatic mutations from two curated databases: The Cancer Genome Atlas (TCGA)^10^, and the International Cancer Genome Consortium (ICGC)^11^. Many of the competing methods evaluated here have been trained in part using 1000 Genomes data, so we use that resource to provide negative examples for our tests. For non-coding regions, we compare *CScape* with scores from FunSeq2 that was designed specifically for predicting oncogenic mutations in non-coding regions^21^. In addition, we report results for methods capable of predicting pathogenic mutations in both coding and non-coding regions: FATHMM-MKL^1^, CADD^7^ and DANN^8^. We evaluate these methods on a population of randomly-selected test sets and compute the average balanced accuracy, area under ROC curve (AUC), sensitivity, specificity, Matthews correlation coefficient (MCC) and positive predictive value (PPV, see Supplementary for definitions). Testing on randomly selected sets allows us to estimate the variation in performance for each method and to compare methods in a disciplined manner. As a control to establish baseline statistics, we use the *CScape* scores for each test set and shuffle the labels. To evaluate the other methods, we apply different thresholds to select positive or negative predictions (see *Score thresholds* in the Supplementary for full details).

Few oncogenic single-point mutations have been verified in non-coding regions. The most prominent to date are three mutations in the *TERT* promoter region^20, 28, 29^. These have been characterised as disruptions to putative ETS (E26 transformation specific) family transcription factor binding sites, along with five additional mutations in *SDHD* and *PLEKHS1* ^20^. The three mutations specific to the *TERT* promoter region are part of the *CScape* training set, and hence one would expect them to be correctly predicted as oncogenic, but the remaining five mutations were previously unseen. For this small number of documented driver mutations, the *CScape* non-coding predictor yielded positive predictions for all but one, (Table 2). This one exception is notable as the only one of the eight whose mutation falls outside the core response element^20^.

**Table 2:** Tests on verified cancer drivers from non-coding regions show that *CScape* predicts all but one.

Gene	Location	Mutation	CScape	FunSeq2^†^	FMKL	CADD	DANN
*TERT*	5:1, 295, 228	G > A	+ (0.52)	+ (1.33)	+ (0.97)	+ (0.34)	+ (0.80)
*TERT*	5:1, 295, 229	G > A	+ (0.62)	+ (1.69)	+ (0.96)	+ (0.66)	+ (0.86)
*TERT*	5:1, 295, 250	G > A	+ (0.58)	+ (0.56)	+ (1.00)	+ (0.31)	+ (0.56)
*SDHD*	11:111, 957, 523	C > T	+ (0.81)	+ (1.00)	+ (0.93)	+ (1.64)	+ (0.99)
*SDHD*	11:111, 957, 541	C > T	+ (0.67)	+ (1.62)	− (0.22)	+ (0.82)	+ (0.85)
SDHD*	11:111, 957, 544	C > T	− (0.40)	+ (1.00)	− (0.14)	+ (0.64)	+ (0.93)
*PLEKHS1*	10:115, 511, 590	G > A	+ (0.65)	− (0.17)	− (0.18)	− (−0.10)	+ (0.53)
*PLEKHS1*	10:115, 511, 593	C > T	+ (0.71)	− (0.17)	− (0.19)	− (−0.06)	− (0.34)

For each method we present the predicted label with its associated score in parentheses. The *CScape* training set includes examples from the *TERT* promoter, hence we expect them to be correctly classified. The remaining examples from *SDHD* and *PLEKS1* promoters are not in the *CScape* training data. DANN is the only other method that yields comparable performance; CADD and FunSeq2 mis-classify both *PLEKHS1* mutations, while FATHMM-MKL (FMKL) mis-classifies the *PLEKHS1* mutations plus two of the *SDHD* mutations. *This mutation in *SDHD* falls outside the putative ETS binding motif. ^†^For FunSeq2 we used a threshold of 0.56 as described in the Supplementary.

The other methods tested also predict a majority of these mutations correctly. For CADD scores we associate negative (−) and positive (+) predictions with negative and positive scores, respectively. For the other methods, we associate negative predictions with scores below 0.5 and positive predictions for the rest. DANN performs as well as *CScape* on these examples, mis-classifying just one of the mutations. CADD and FunSeq2 mis-classify two mutations, both from the *PLEKHS1* gene. FATHMM-MKL (FMKL) mis-classifies the *PLEKHS1* mutations as well as two of the *SDHD* mutations.

#### International Cancer Genome Consortium (ICGC) data

**Figure 4:**
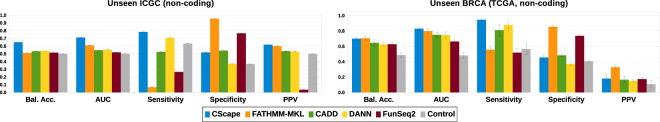
CScape yields state-of-the-art performance on unseen examples in non-coding regions. **Left:** On the ICGC test set, *CScape* significantly outperforms competitors for all performance measurements except specificity. **Right:** On the BRCA test set *CScape* yields balanced accuracy (70%) and AUC (0.84) that are similar to its nearest competitor (FATHMM-MKL, 71% and 0.80, respectively). Error bars indicate a 99% confidence interval for statistics measured across 20 test sets. ^†^For FunSeq2 we report results at its *peak* balanced accuracy threshold, as described in the Supplementary.

Within the ICGC data, we found 1,633,449 examples for testing in non-coding regions after we applied our strict filtering criteria and removed examples found in our non-coding training data. For our negative examples from the 1000 Genomes dataset we used examples within 1,000 positions of a positive example and removed the remainder from our training set. This yielded more than 3 million examples, so to achieve a balanced test set, we sampled 1,633,449 of these for our test set. Hence the final ICGC test set contains 3,266,898 examples. *CScape* yields significantly higher balanced accuracy than competing methods on these data (*p* < 10^−7^, Mann-Whitney) even at the default threshold (Fig. 4 (top), Supplementary Table S7). Accuracy at the default threshold is 65%, while FATHMM-MKL yields 54%. Other metrics are similarly strong, with the exception of specificity. At the cautious classification threshold, all statistics improve considerably, with balanced accuracy and AUC scores well over 80%, whilst yielding predictions for 640,312 test examples (19.6%).

#### The Cancer Genome Atlas (TCGA) breast cancer dataset

Within the TCGA database we found a small set of 3,368 examples in non-coding regions having a *Valid* status. The vast majority of these appear in our training set, but 360 examples, all associated with breast cancer (BRCA), were previously unseen. For non-coding tests, we select neutral examples from the 1000 Genomes database in the same way as we did for the ICGC data, by using examples that fall within 1,000 positions of some positive example. After removing examples found in our training set, we had a test set with 3,206 examples (360 positive, 2,846 negative). We compared the *CScape* non-coding predictions with those of four other methods relevant to non-coding examples: our FATHMM-MKL, CADD, DANN and FunSeq2. On these data *CScape* at the default threshold is competitive with current state-of-the-art, with average balanced accuracy comparable to FATHMM-MKL and higher than other methods (Fig. 4 (bottom), Supplementary Table S8). At the cautious classification threshold *CScape* yields higher scores than competitors in all categories except specificity, whilst producing predictions for 795 examples (24.8%).

### Evaluation on previously unseen coding examples

To evaluate our models on unseen examples in coding regions, we downloaded putative somatic mutations from two additional curated databases: the Database of Curated Mutations (DoCM)^30^, and ClinVar^31^. The DoCM represents a refined subset of examples from the other databases, hence we remove the DoCM examples from the other test data so that we may treat each data set independently. For coding regions, we compare *CScape* with scores from TransFIC^27^, which provides scores from MutationAssessor (MAS)^6^, PolyPhen2^4^ and SIFT^5^ that have been optimised for predicting oncogenic mutations. As we did in non-coding regions, we also report results for general-purpose predictors FATHMM-MKL, CADD and DANN.

#### Database of Curated Mutations (DoCM) data

**Figure 5:**
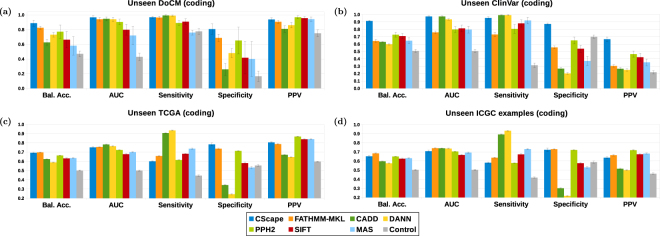
CScape exhibits strong performance on unseen examples in coding regions. (**a**) For 724 unseen DoCM examples, *CScape* tends to outperform other methods, with balanced accuracy over 80% averaged over 10 test sets, compared with 75% for FATHMM-MKL, its nearest competitor. **(b)** For 5,642 unseen ClinVar examples, *CScape* yields significantly higher values than other methods for all statistics except AUC and Sensitivity. **(c)** For 108,874 unseen TCGA examples, *CScape* yields balanced accuracy that is competitive with its closest competitor, FATHMM-MKL. **(d)** For 92,252 unseen ICGC examples, *CScape* yields slightly lower balanced accuracy than its top competitor (65% versus 68% for FATHMM-MKL), while PolyPhen2 yields much higher average PPV. Error bars indicate a 99% confidence interval for statistics measured across 20 test sets.

The Database of Curated Mutations (DoCM) represents a carefully curated set of 1,364 somatic mutation examples, including 1,223 distinct SNVs^30^. Of these, 674 SNVs appeared in our training set and 551 were previously unseen. To create a test set, we found 289 germline mutations in the 1000 Genomes database within 10,000 positions of some DoCM example, of which 173 were absent from the *CScape* training set. This comprised an unbalanced test set we used to compare *CScape*’s predictions with other classifiers for coding-region cancer mutations (Fig. 5(a), Supplementary Table S9). Given the relatively small data set, we used *N* = 10 test samples to generate statistics. At the default threshold, *CScape*’s balanced accuracy averaged over the 10 sets is 88%, significantly higher than its closest competitor, FATHMM-MKL, at 83% (*p* < 0.012, Mann-Whitney). At the cautious classification threshold (*p*-scores below 0.11 or above 0.89) *CScape* makes predictions for a total of 275 examples (38% overall), but only eight of these are negatives, four of which yield false-positives. This severe imbalance yields extremely low balanced accuracy (see Fig. 3) and high variance for any combination of test sets, but sensitivity is consistently 100% and the PPV is 99%, providing evidence that *p*-scores above this threshold correctly identify oncogenic examples.

#### ClinVar

The ClinVar database contains both germline and somatic variants, hence we used annotations to identify somatic mutations. Within its extensive metadata, ClinVar mutations include a *clinical significance* (CLNSIG) that signifies the likelihood that a mutation is pathogenic, and a *variant disease name* (CLNDBN) that we use to identify somatic variants. For our test set, we selected SNVs with a CLNSIG designation of *pathogenic* or *likely pathogenic* for our positive examples, and those with a *benign* or *likely benign* designation as negatives. From these, we selected examples with a CLNDBN containing *tumor* or *cancer* designations and filtered out any examples found in our model’s training set. This yielded an unbalanced set of 5,642 examples (1,186 positive, 4,456 negative) for testing.

Results (Fig. 5(b), Supplementary Table S10) show that *CScape* at the default threshold yields significantly higher average balanced accuracy, PPV and MCC values than the other methods (*p* < 10^−7^, Mann-Whitney), and a significantly higher average AUC (*p* < 10^−6^) than all methods except CADD. The cautious classification threshold yields similarly strong results, but with significantly higher MCC and PPV even than *CScape* (*p* < 10^−7^, Mann-Whitney) at the default threshold.

#### The Cancer Genome Atlas (TCGA) data

TCGA data includes a *validation status* we use to remove low-confidence examples. For our test set, we retained 66,441 out of 4.2 million TCGA examples with a *Valid* status. Of these, we removed 1,898 that were in the *CScape* training set, leaving 64,543 positive examples. As in our other tests, we selected neutral examples from the 1000 Genomes database, using examples that fall within 10,000 positions of some positive example. Of 41,319 examples selected from 1000 Genomes, we removed 9,835 that were also in the *CScape* training set. This left 31,484 negative examples in our test set, for a total of 109,085.

We compared *CScape*’s performance on the TCGA data with six other methods relevant to coding-region predictions (Fig. 5(c), Supplementary Table S11). At the default threshold (*τ* = 0.5), *CScape* yields higher balanced accuracy, MCC and specificity scores than the other methods on these data. In addition, *CScape’s* PPV and AUC scores are competitive with the best alternatives. CADD and DANN, modeled from the same training set, yield the highest sensitivity but the lowest specificity. At the peak threshold *τ* = 0.89 established for cautious classification, *CScape* makes confident predictions for 17,801 test examples (16.3%) and yields the highest performance statistics in all categories.

#### International Cancer Genome Consortium (ICGC) data

We downloaded 61 mutation files from ICGC containing 47 million distinct single-nucleotide variants (SNVs). ICGC annotates mutations using four different verification statuses: *tested and verified*, *tested and verified to be false*, *tested and inconclusive* and *not tested*. For positive test examples we selected those having *tested and verified* for both *verification* and their *biological validation* statuses, and we required that examples were shared by at least two distinct donors (*donor id*). After removing examples found in our coding-region training data, we had 42,515 positive examples. We found just 106 distinct ICGC SNVs with the annotation *tested and verified to be false*, but none of them met our filtering criteria. Hence as in other tests, we selected as a negative test set examples from 1000 Genomes within 10,000 positions of some positive example and removed those found in our training set. Our final ICGC test set contains 92,732 examples (42,174 positive, 50,558 negative).

For these unseen ICGC examples we find that *CScape* using the default threshold (0.50) is competitive with the other methods tested, though on these data it slightly under-performs its top competitor, FATHMM-MKL (Fig. 5(d), Supplementary Table S12). In particular, the cancer-optimised PolyPhen2 scores yield an exceptionally high average PPV in our tests. With cautious classification threshold, *CScape* yields substantially higher balanced accuracy, MCC, PPV and AUC scores than all other methods, producing predictions for 13.1% of examples.

#### Mis-classified test examples

Despite *CScape’s* strong performance on both coding and non-coding test examples, we investigated mis-classified examples to see if patterns emerged. To this end we used the Variant Effect Predictor (VEP) to identify the regions that may be different between correctly classified and mis-classified test examples (Supplementary Figures S8–S9). We first note that any mutation may impact multiple transcripts, hence the same mutation may have multiple annotations in the VEP database. For our coding classifier, we find the majority of the 627 mis-classified ClinVar examples are annotated as missense mutations for at least one transcript (Supplementary Figure S8). while the majority of correctly classified examples are annotated as synonymous. In our other test sets, there appear to be no strong trends though we do find a slightly higher proportion of correct predictions amongst mis-sense mutations. The non-coding classifier shows little sign of classifying one kind of example better than another (Supplementary Figure S9). On the relatively small BRCA test set (3,206 examples) the model tends to mis-classify examples designated *NC transcript* and *Noncoding exon* at a slightly higher rate than others, but on the larger TCGA test set (3.3 million examples) the model shows no preference for any particular gene characteristic.

### Cancer-specific thresholds for competitors

Using the default thresholds for all methods, we find that *CS-noncoding* outperforms competitors on all of our benchmark tests, and that *CS-coding* either outperforms competitors or yields comparable balanced accuracy. However, several of these other methods (notably CADD, DANN and FATHMM-MKL) have not been optimised for oncogenic mutations. Therefore, we used our training data to identify alternative thresholds for all competing methods across their prediction domains (coding, non-coding or genome-wide) and assessed the potential change in their performance (Supplementary Methods). Overall, results were mixed, with CADD and DANN improving marginally in all regions, while FATHMM-MKL generally performed worse. We also identified region-specific thresholds using either coding or non-coding training sets, and found significant improvements for both CADD and DANN. Hence it may be possible to apply non-cancer-specific methods to cancer variants, provided one can establish appropriate thresholds. However, we believe this requirement would be onerous for most users, and reiterate that *CScape* achieves similar or higher accuracy at its default threshold.

### Regions of Interest

Our Regions of Interest method highlighted multiple segments within the exons of genes as potentially functional as disease-drivers. Since our classifiers were trained on COSMIC data across multiple cancer types, highlighted genes tend to be of broader significance across multiple tumour types, such as the tumour suppressor gene *TP53*, rather than genes with known associations with a particular type of cancer. We provide files listing these regions of interest, in both coding and non-coding regions, at the CScape website.

## Discussion

A stated top balanced test accuracy of 91.7% in coding regions has been made possible through use of a broad set of data sources, with subsequent use of cautious classification to define a more accurate predictor, but with more limited applicability (this accuracy can only be achieved at 17.7% of nucleotide positions). Without cautious classification, *CScape* yields competitive accuracy in both coding and non-coding regions, but its performance in non-coding regions excels in our tests when compared with other methods. We note that both models, *CS-coding* and *CS-noncoding*, benefit from similar but distinct feature groups. In particular, *CS-noncoding* benefits from the *Mappability* group that measures the uniqueness of a region, possibly allowing the model to learn the relative oncogenic susceptibility of repeat regions or paralogous sequences. Predictably, *CS-coding* benefits largely from predicted amino acid substitutions and sequence conservation measures. The positive predictive values (PPV) and numbers of true positives predicted (Table 1) suggest that accurate prediction of positives is achievable, and that the balanced test accuracy is not overwhelmingly achieved through accurate prediction on neutral variants. Currently the paucity of validated oncogenic variants in non-coding regions is a limiting factor in creating accurate predictors for those regions; as more of these examples are confirmed, subsequent models should improve. In addition, it should be possible to improve on these results across the entire cancer genome, via identification of further sources of data relevant to predicting disease-driver status, the use of adaptive recurrences thresholds *r* in different regions of the coding and non-coding parts of the genome, and the development of cancer-type specific predictors e.g. for breast cancer or prostate cancer (in previous work^32^ we have established this leads to improved balanced test accuracy). Similarly, we have shown that some competing methods may benefit from using cancer-specific thresholds, given an appropriate data set for evaluating their accuracy at different thresholds. However, it is doubtful that users will be willing to engage in extensive post-processing, and the resulting predictions are unlikely to yield balanced accuracy significantly better than *CScape’s*.

One of our goals in this study was to exploit the diverse databases emerging from the ENCODE resource. Many of these databases are sparse: some of these feature groups have values for only a tiny proportion of locations across the genome. In this study we explored two data integration methods that work well with whole feature groups: data-level integration, in which we merge groups into a single kernel, and kernel-level integration in which we treat each group as a separate kernel and use MKL to combine them. However the sparsity of many feature groups currently limits their utility in these paradigms. This sparsity could be due to the constraints of the underlying assays, such as expression data obtained for specific conditions, or due to data that are inherently sparse, such as transcription factor binding sites. Regardless of the cause, more sophisticated data integration methods will likely be required to exploit them fully.

As this paper was in preparation, evidence emerged demonstrating that a substantial number of the examples in mutation databases are probably spurious, reflecting damaged DNA rather than mutations^33^. We mitigate this issue to some extent by using recurrence counts or validation status to filter positive examples, but negative examples could be more severely impacted. Hence we should view the statistics reported here with some scepticism, as the models we have examined have been trained and tested on putative oncogenic driver mutations and, possibly, a mixture of benign mutations and damaged DNA. Only once spurious examples have been largely eliminated from mutation databases will we know whether the added noise renders the discrimination problem more or less difficult than reported here.

## Methods

### Model selection

All experiments were written in Python (v2.7.6) using classification models from *PyML* ^34^ (v0.7.13.3) and *scikit-learn* ^35^ (v.0.17). We use LOCO-CV to evaluate a variety of different classification models, to establish the best overall model, using balanced accuracy as our criterion. In each CV fold, we select a training set at random from 21 chromosomes, hence performance will change from one run to the next. For this reason, we ran cross validation 30 times for each model, and use the average balanced accuracy to compare models. Many classifiers have parameters that require tuning, such as a regularisation parameter. To find the best parameter for each model, we selected a range of values (*C* = 10^−4^ to 10^4^, in the case of SVM) and ran LOCO-CV 10 times for each value, selecting the parameter that yielded the best average balanced validation accuracy for the model (see Supplementary text and Supplementary Table S5 for more details). We found that Gradient Boosting^36^ yielded the strongest performance overall, though several models yielded balanced accuracy nearly as high (Supplementary Table S5). In particular, support-vector machines with Gaussian kernels were competitive, facilitating experiments with multiple kernel learning (MKL).

### Establishing thresholds

Our classifiers produce scores for every possible mutation, where scores may take on any value in ${\mathbb{R}}$. A common approach for binary classification is to apply a threshold of 0, and predict values below the threshold as negative and those above as positive. An alternative method is to map output scores to a probability distribution on [0, 1] and use a threshold of 0.5. In this work we use Platt scaling^37^ to associate a confidence measure with each predicted class label, which we interpret as the probability that a particular SNV is a cancer driver. By establishing a suitable confidence threshold we can also restrict prediction to a subset of higher balanced-accuracy predictions (*cautious classification*). This leads to an alternative, high-confidence threshold for our models that yields improved balanced test accuracy, but at the expense of prediction on a subset of nucleotide positions.

### Regions of interest

The resultant positive (oncogenic) predictions are frequently clustered by genomic location. Thus mutations in regions that are highly conserved across species are typically more deleterious than mutations in regions exhibiting high inter-species variability: the motivation for using sequence-conversation features for the classifier. This fact alone should give rise to significant clustering of positive predictions in highly conserved regions. To isolate regions of clustered positive predictions, which we call *Regions of Interest* (*RoI*s), we determined the distribution of confidence values and labeled a region as a *RoI* if the contiguous set of positive confidence values located in this region had a very low expectation of being observed, relative to the overall distribution. Full details on the methods used to identify these regions are given in the Supplementary Methods.

### Data availability

*CScape* predictions are available as an online resource http://CScape.biocompute.org.uk. In addition, a predictions database, software for querying the database, and training and test datasets are all available for download from the website.

## Electronic supplementary material

Supplementary Information
