# Supplementary material for: CScape: a tool for predicting oncogenic single-point mutations in the cancer genome
Source: Sci Rep. 2017 Sep 14;7:11597. doi: 10.1038/s41598-017-11746-4 (PMC5599557; doi:10.1038/s41598-017-11746-4)
Supplement: Supplementary file 1 — Supplementary Information [file 41598_2017_11746_MOESM1_ESM.pdf]

Supplementary Information for

CScape: a tool for predicting oncogenic single-point mutations in the cancer genome

Mark F. Rogers[1,*], **Hashem A. Shihab**[2], **Tom R. Gaunt**[2], **and Colin Campbell**[1]

[1]Intelligent Systems Laboratory, University of Bristol, Bristol, BS8 1UB, United Kingdom
[2]MRC Integrative Epidemiology Unit (IEU), University of Bristol, Bristol, BS8 2BN, United Kingdom
[*]Mark.Rogers@bristol.ac.uk

Data selection

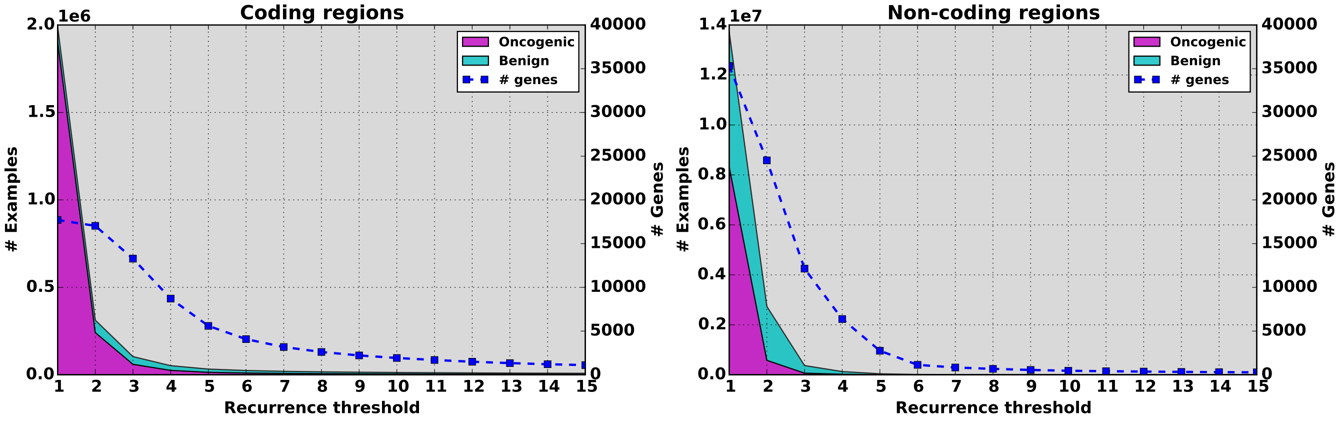

Supplementary Figure S1. The number of COSMIC examples available for training decreases dramatically as we increase the recurrence threshold. Positive examples (*Oncogenic*, magenta areas) show the most dramatic drop when we increase recurrence threshold r from $r = 1$ to $r = 2$ in coding regions (left) or non-coding regions (right). Neutral examples (cyan areas) also decrease as they are confined to regions near positive examples. The number of genes represented also decreases, but less rapidly (blue squares). Note that there are more genes shown for non-coding regions, as these include non-coding gene types such as snRNA, miRNA, lincRNA and pseudogenes.

We constructed our pathogenic (positive) dataset using somatic point mutations from the COSMIC database[1] (version 75, November 2015) while we used SNVs from the 1,000 Genomes Project[2] as putative non-drivers. To increase our confidence in the putative drivers in our training set, we wish to select examples with the highest recurrence levels possible. However, we balance this against the amount of data available for training, along with any potential bias that may occur by restricting the number of examples too severely. As we increase the minimum recurrence level, r, the number of training examples drops dramatically (Figure S1). We choose as our threshold the highest recurrence level that shows little evidence of bias and provides sufficient training examples to create an accurate classifier. To detect bias, we filter training examples using increasing values of r. We then run LOCO cross-validation using the top-performing model selected via sequential learning (see *Data-level integration*. and record the average balanced accuracy over 30 runs, using balanced training sets of 1,000 examples per fold (see Figure S2). There are two indicators of potential bias: first, average balanced accuracy may increase significantly, indicating that the reduced test sets have become easier to predict; second, variance over the 30 runs may increase, suggesting that balanced accuracy will depend on the training data and hence over-fitting becomes a concern. We choose our value of r using the maximum value of r that shows no significant increase in balanced accuracy and no noticeable increase in variance. For coding models, we choose $r = 5$ and for non-coding, $r = 3$.

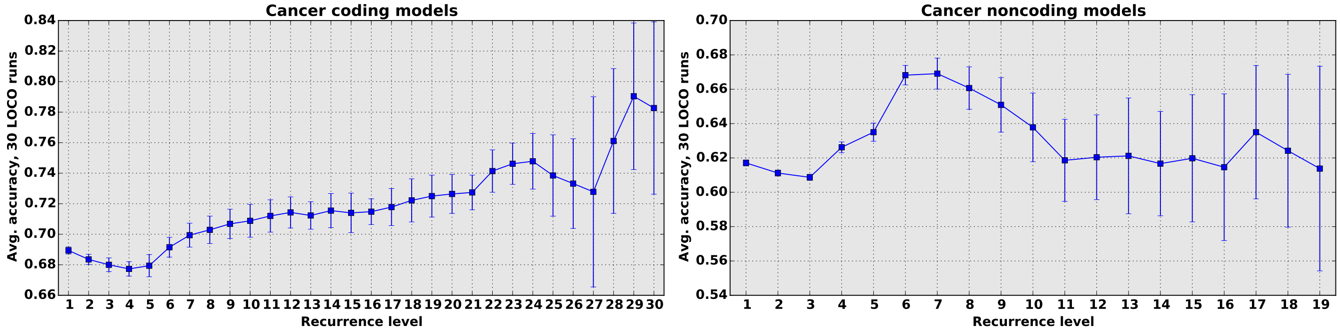

Supplementary Figure S2. Accuracy as a function of recurrence level for cancer coding models (left) and noncoding models (right). For both coding and noncoding models, balanced accuracy at $r = 1$ is slightly higher than for $r = 2$ or $r = 3$. When the minimum recurrence value rises to $r \geq 6$ for coding models, or $r \geq 4$ for noncoding models, a rapid increase in balanced accuracy suggests possible bias as we confine our training set only to high-recurrence SNVs. Accuracy reaches a nominal peak at $r = 24$ for coding models and $r = 6$ for noncoding models, beyond which variability increases markedly as the amount of data available for training and testing decreases. Accuracy was computed using LOCO cross-validation; gradient boosting models were all trained on $N = 1,000$ examples (500 positive/500 negative) using 16 estimators in all cases.

Bias may also be introduced if non-driver mutations are located in genomic regions that differ substantially from regions containing driver mutations, such as examples from different genes. To ensure the locations of putative non-driver mutations approximate those of driver mutations, we select only those putative non-drivers found within a window w of a putative driver mutation. To this end, we use the same procedure outlined above: at different levels of w we run LOCO cross-validation for 30 runs and record the average balanced accuracy over these runs. We then choose our value of w using the maximum value that shows no significant increase in balanced accuracy and no noticeable increase in variance. For coding regions we choose $w = 10^4$ and for non-coding regions, $w = 10^3$ (Figure S3).

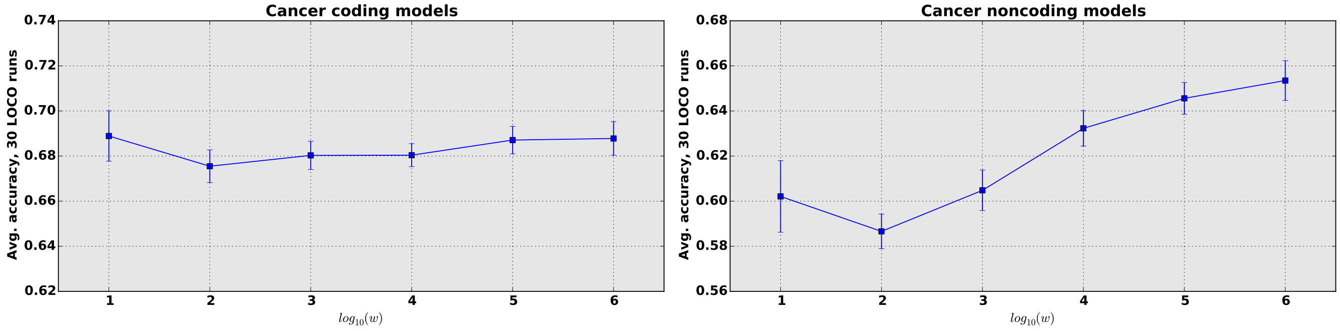

Supplementary Figure S3. Accuracy as a function of neutral example window size for cancer coding models (left) and noncoding models (right). To ensure that neutral examples and positive examples all come from similar genomic regions, we require all neutral examples to fall close to some positive example, within a window of w positions. These graphs show the effect on balanced accuracy of window sizes from $w = 10^1$ to $w = 10^6$. Accuracy for coding-region models remains consistent as we increase the window size, while models for noncoding regions may become biased when $w \geq 10^4$. Accuracy was computed using LOCO cross-validation; gradient boosting models were all trained on $N = 1,000$ examples (500 positive/500 negative) using 16 estimators in all cases.

Chromosome	Coding		Non-coding	
	COSMIC	1000G	COSMIC	1000G
1	1,463	3,506	5,760	5,427
2	897	1,847	5,888	5,436
3	1,194	1,373	4,567	4,358
4	565	1,026	4,365	4,574
5	580	1,228	4,310	4,129
6	812	2,137	3,446	3,820
7	790	1,401	4,455	4,490
8	470	1,003	3,970	3,992
9	596	1,234	2,441	2,226
10	495	1,028	3,258	3,460
11	905	2,365	3,300	3,445
12	727	1,339	2,689	2,609
13	247	370	1,935	2,044
14	344	1,077	2,039	1,926
15	408	888	1,902	1,832
16	506	1,760	2,522	2,593
17	1,321	1,818	1,821	1,753
18	180	365	1,601	1,691
19	1,107	4,215	1,704	1,843
20	339	817	1,611	1,591
21	163	451	1,530	1,257
22	280	783	972	1,132
Total	14,389	32,031	66,086	65,628

Supplementary Table S1. Distributions of training examples by chromosome shows the number of examples available for testing and training in LOCO cross-validation. These data are unbalanced, with 2 to 5 times as many neutral (1000G) examples as positive (COSMIC) examples.

Features

Conservation and ENCODE data

As in previous studies[3,3,4] we evaluated over 30 distinct ENCODE datasets as potential feature groups for these classifiers. Broadly speaking, we divide these datasets into eight categories:

- *Genomic and Evolutionary*: where appropriate, we used a number of genomic properties such as gene length, number of transcripts and the average number of predicted protein domains across transcripts. In addition, we used a comprehensive set of conservation-based measures, such as dN/dS ratios between human and 65 different species (one-to-one orthologues). We also used several conservation based measures, e.g., PhyloP[5] and PhastCons[6] scores, derived from the multiple sequence alignment of 46 and 100 vertebrate genomes to the human genome[7].

- *Histone Modifications*: we used ChIP-Seq peak calls for 14 histone modifications across 45 cell lines from ENCODE[8] and narrow, broad and gapped regions of enrichment based on consolidated epigenomes from the NIH Roadmap project[9].

- *Open Chromatin*: we used DNase-Seq and Formaldehyde-Assisted Isolation of Regulatory Elements (FAIRE) peak calls across 119 cell lines from ENCODE and narrow, broad and gapped regions of enrichment based on consolidated epigenomes from the NIH Roadmap project.

- *Transcription Factor Binding Sites*: based on PeakSeq and SPP peak calls for 119 transcription factors across 77 cell lines from ENCODE.

- *Gene Expression*: based on RNA-seq signal coverage using consolidated epigenomes from NIH Roadmap Epigenomics.

- *Methylation*: based on whole genome bisulphite sequencing (WGBS) from NIH Roadmap Epigenomics.

- *Digital Genomic Footprinting Sites*: for transcription factor recognition sequences within DNase-hypersensitive sites using consolidated epigenomes from the NIH Roadmap Epigenomics Project.

- *Networks*: we used measures of centrality from cell-type specific interactome and tissue-specific co-expression networks.

We found just four of the ENCODE feature groups (Table S4, bold values) relevant to somatic mutations in noncoding regions, while none of these groups yielded good discrimination in coding regions. One reason may be the sparse coverage of these data: we found that many examples had no corresponding data in these feature groups. This sparsity limits the information available for training our models and for making novel predictions, and so can undermine the performance of classifiers that rely on these data[4].

Genomic context features

For our coding classifier we also include features that describe the genomic context where a mutation occurs. For coding regions we base these features on information from the ENSEMBL Variant Effect Predictor (VEP). The VEP provides characteristics for specific genomic locations that we can exploit to predict the likely impact of a SNV. These may include transcript features and amino acids impacted by a mutation, relative allele frequencies, and scores from pathogenic variant predictors such as SIFT and PolyPhen[10]. To mitigate potential bias we are careful not to include these other scores, nor do we include features such as PubMed IDs that may have been used to curate SNV databases. Hence our features include only the following elements:

- *Consequence:* the VEP provides these as annotations of 35 types of changes to associated transcripts, such as *3' UTR variant*, *missense variant* or *TF binding site variant*. We represent these using 35-element binary vectors (one bit per annotation), plus a count of the number of transcripts possibly impacted. Note that we do not encode the impact levels provided on the VEP website (HIGH, MODERATE, MODIFIER, LOW); instead we allow our model to learn priorities in training.

- *Amino acid:* the amino acids inferred by the reference and allele nucleotides. To capture the change in amino acid composition, we construct two sets of features: two 20-element binary vectors that reflect the reference and allele residues, respectively, and two real-valued vectors that represent specific residue characteristics: molecular weight, hydrophobicity, occurrence frequency, dissociation constants for the $COOH$ and NH_3^+ groups, and the pH at the isoelectric point.

We apply the VEP features only to our coding predictor, as amino acid features are not relevant to SNVs in non-coding regions, and there are far more non-coding positions than coding positions: such a vast number of VEP queries would be impractical for creating a genome-wide database. For non-coding SNVs we employ a simpler approach by measuring the proximity of each SNV to gene features such as the transcription start site (TSS), splice sides, and codons. In this method we establish a window w around each mutation and count the annotated features that fall within that window, or measure the distance to each feature (Supplementary Table S3).

Spectrum features

Our models should learn the sequence characteristics that are most susceptible to oncogenic mutations in both coding and non-coding regions. As a simple way to capture the disruption that may occur in the sequence surrounding a mutation, we use *spectrum* kernels[11] to compare the composition of k-mers within a region before and after a mutation is applied to a sequence. Given a mutation and its flanking sequence, we obtain the k-spectra for the wild-type and mutated versions of the sequence and concatenate these features to provide a picture of the region before and after mutation. Formally (borrowing notation from[11]), if the k-spectrum of an input sequence s is the set of all k-length contiguous subsequences, then we define a feature map of all possible subsequences a of length k from alphabet $\mathscr{A}$ as follows:

$$\Phi_k(s) = (\phi_a(s))_{a \in \mathscr{A}^k} \tag{1}$$

where $\phi_a(s)$ is the count of the number of times sequence a occurs in sequence s (from which a kernel matrix can be readily derived[12]). We found that these features perform competitively on their own, and improve balanced prediction accuracy in our merged-kernel tests (Supplementary Figures S6-S7). As this approach makes no assumptions about the kind of RNA binding proteins that may be impacted by a particular mutation, it eliminates the requirement to find and assess known motifs.

For these features we optimise two relevant parameters: the size of the window w flanking each mutation, and the maximum k-mer size, k. For a single-point mutation we expect the disruption to be confined to a relatively small region around the mutation. This restricts the useful window size and in turn, the maximum k-mer sizes that will be relevant. For both coding and non-coding models we performed a grid search over these sizes (Table S2). In both cases we found that a window size $w = 3$ and maximum k-mer size 2 yielded the best results: 59.5% balanced accuracy for the coding model and 56.9% for the non-coding model.

	Maximum k-mer size							
	coding				non-coding			
Window	$k=1$	$k=2$	$k=3$	$k=4$	$k=1$	$k=2$	$k=3$	$k=4$
$w=1$	0.54	—	—	—	0.57	—	—	—
$w=3$	0.58	**0.60**	0.59	—	0.56	**0.57**	0.56	—
$w=5$	0.59	0.59	0.59	0.59	0.54	0.56	0.56	0.55
$w=7$	0.58	0.58	0.59	0.58	0.53	0.55	0.55	0.55
$w=9$	0.58	0.58	0.58	0.58	0.53	0.55	0.55	0.54

Supplementary Table S2. Spectrum kernel performance in coding regions using different values for window size w and maximum k-mer size k reveals that the highest average balanced accuracy occurs at a window size of 3 and maximum k-mer size of 2 for both coding (60%) and non-coding (57%) models (values in **bold**). Accuracy was averaged over 30 LOCO-CV runs with a balanced training set size of N=2,000 for each fold.

Variant Effect Predictor

We concatenate two vectors to encapsulate all of the Variant Effect Predictor (VEP) features used in the CScape model for coding regions. The *Consequences* features consist of a transcript count (the number of transcripts that may be impacted by a mutation) and a vector of binary flags that represent the possible consequences returned by the VEP (Table S3). The *Amino acids* features consist of two vectors that encapsulate the possible amino acids associated with each mutation. The first amino acid vector represents the amino acids associated with the wild-type sequence, and the second represents the amino acids associated with the mutation. Instead of using a simple binary flag, we use counts to record the number of transcripts impacted by each amino acid change.

transcript ablation	*splice region variant*	*upstream gene variant*
splice acceptor variant	*incomplete terminal codon variant*	*downstream gene variant*
splice donor variant	*stop retained variant*	*TFBS ablation*
stop gained	*synonymous variant*	*TFBS amplification*
frameshift variant	*coding sequence variant*	*TF binding site variant*
stop lost	*mature miRNA variant*	*regulatory region ablation*
start lost	*5 prime UTR variant*	*regulatory region amplification*
transcript amplification	*3 prime UTR variant*	*feature elongation*
inframe insertion	*non coding transcript exon variant*	*regulatory region variant*
inframe deletion	*intron variant*	*feature truncation*
missense variant	*NMD transcript variant*	*intergenic variant*
protein altering variant	*non coding transcript variant*	

Supplementary Table S3. Consequence codes encapsulated in the VEP features. The coding region classifier uses a binary vector to identify which of these consequences is annotated for a particular mutation.

We find that both sets of VEP features perform similarly (Figure S4). In LOCO cross-validation using balanced training sets of 2,000 examples for each chromosome, the *Consequences* features yield 66.0% balanced accuracy on average, while the *Amino acids* features yield 65.5%. When we combine these features we see a slight improvement overall, up to 67.4%, making it the most accurate group used for the coding region classifier (Table S4).

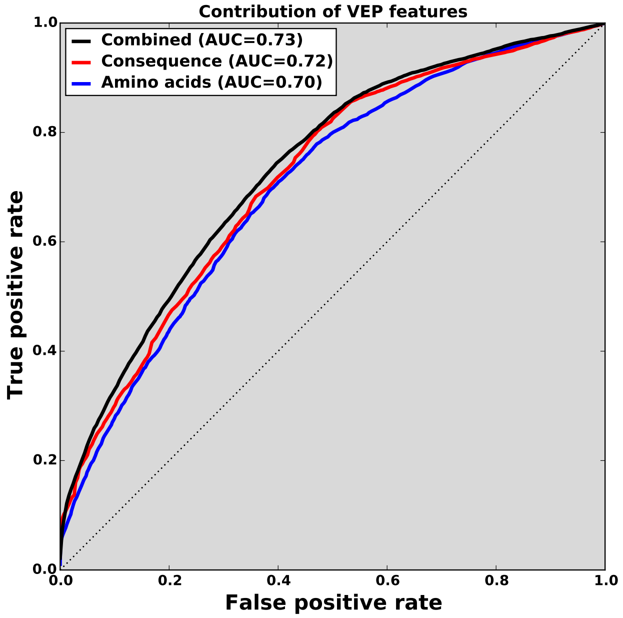

Supplementary Figure S4. ROC curves depict the contribution of distinct VEP feature sets in coding regions. The amino acid features encapsulate the possible amino acids represented by the wild-type and mutant sequences. Where a mutation falls within multiple transcripts several amino acids may be reported. The consequence features encapsulate locations within a gene that may be impacted by a mutation. Both sets of features are informative on their own; however the combination of the two yields slightly better performance.

Distance features

As noted in the main text, we apply the VEP features only to our coding predictor, since amino acid features are not relevant to SNVs in non-coding regions, and there are far more non-coding positions than coding positions. For non-coding SNVs we employ a related, but simpler approach: we measure the distance from each SNV to gene features annotated in the ENSEMBL gene models: *start codon*, *stop codon*, *gene*, *UTR*, *CDS* and *exon*. This approach is simple, yet should enable our models to learn relationships between SNVs and important gene elements. For example, exon boundaries help to identify mutations close to splice sites. Similarly, 5' gene boundaries identify mutations close to transcription start sites or promoter regions. To capture this information, we establish a window w around each mutation and either count each of the elements that fall within the window, or measure the distance to the nearest example of each element. We encapsulate counts features using a vector of six integer values. Distance features also comprise six features, but the distance to nearest feature is mapped into the range $[0,1]$ as follows. If a mutation is d positions away from the nearest element, $0 \leq d \leq w$, then the score s is given as:

$$s = \begin{cases} \frac{1}{d+1}, & d < w. \\ 0, & \text{otherwise}. \end{cases} \tag{2}$$

To identify the optimum setting for the window, w, we ran LOCO CV using values from $w = 1$ to $w = 10^6$ and found that $w = 10$ yielded the best performance for both coding and non-coding models. For coding models, the scoring representation given by Equation 2 worked best, while for non-coding models, the counts representation worked best. The balanced accuracy of both models are recorded as *Distance* in Table S4.

Feature groups

We used LOCO-CV to evaluate more than 30 distinct feature groups: two conservation-based groups, the spectrum kernel, two groups designed to encapsulate the genomic context (gene region, any corresponding amino acids, or proximity to gene features), and 27 groups based on ENCODE data (Table S4). Conservation groups yield relatively high balanced accuracy for both non-coding and coding examples, consistent with previous studies showing the importance of conservation in noncoding regions[3,13]. The spectrum kernel scored equally well for non-coding regions, suggesting that it may learn patterns of regulatory

motifs gained or lost through mutation. Genomic context features also appear amongst the top-performing groups. ENCODE features generally perform poorly, due partly to the number of missing values (see Figure S5).

	Balanced accuracy	
Feature group	Non-coding	Coding
Conservation scores		
46-way cons.	**0.572**	**0.655**
100-way cons.	0.569	0.649
Spectrum kernel	**0.570**	**0.597**
Genomic context		
VEP	—	**0.674**
Distance	**0.515**	**0.605**
ENCODE features		
Mappability	**0.514**	0.541
BroadPeak	0.511	0.587
NarrowPeak	0.511	0.567
RNA	0.509	0.540
GappedPeak	0.507	0.575
BroadHMM	0.507	0.565
ChromHMM15	0.507	0.564
LongRnaSeq	0.507	0.553
Segmentation	0.507	0.538
ChromHMM18	0.506	0.567
ChiaPet	0.506	0.545
Repeats	0.505	0.502
RnaChip	0.504	0.542
GC	0.504	0.537
Histone ChipSeq	0.503	0.570
GeneSt	0.502	0.556
Riken	0.502	0.551
ShortRnaSeq	0.501	0.525
PeakSeq	0.501	0.510
TFBS Uniform	0.501	0.501
DNase Uniform	0.500	0.519
FDR peaks	0.500	0.518
RipSeq	0.500	0.512
FAIRE	0.500	0.507
SPP (TFBS)	0.500	0.501
Footprints	0.500	0.500
Tiling	0.500	0.500

Supplementary Table S4. Conservation, spectrum kernel, genomic context and ENCODE feature groups evaluated on COSMIC somatic mutations and 1000 Genomes neutral mutations. All feature groups were evaluated using LOCO cross-validation in which each training set was constructed from a balanced set of 2,000 examples. Feature groups are organized by category and shown in descending order of balanced accuracy for *non-coding* data. Shown in bold are the balanced accuracy statistics for the feature groups used in our final models. While none of the datasets individually yields high balanced accuracy, we find that as in previous studies, conservation scores are among the most informative features. Spectrum kernels also appear to provide some discrimination. In non-coding regions, we find that one of the ENCODE groups, *Mappability* provided enough discrimination power to improve performance in our final model. In coding regions, the ENCODE datasets generally do not perform as well as conservation or spectrum features, while the VEP features yield by far the highest balanced accuracy.

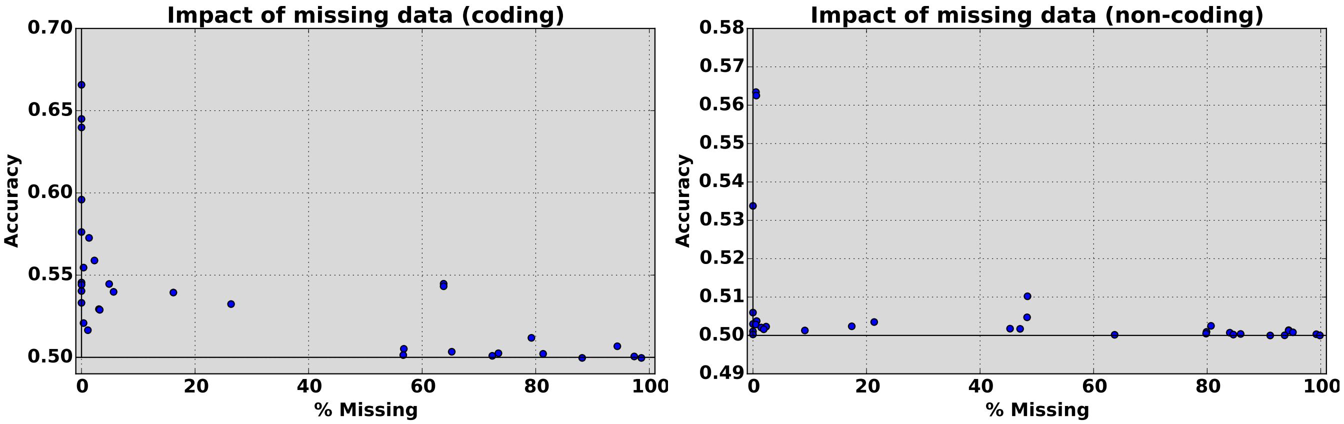

Supplementary Figure S5. Graphs depicting balanced accuracy as a function of the proportion of missing values show a direct correspondence between sparsity, measured as the proportion of missing values in a feature group, and performance, measured as balanced accuracy.

Data-level integration

The simplest kernel method for integrating different data sources is to combine the features from all sources into a single kernel. This allows a model to discriminate between classes by learning how features from one source may interact with features from other sources. Given 16 possible data sources for noncoding regions and 17 sources for coding regions, there were up to 131,000 possible combinations of feature groups, so exhaustive testing was impractical. Instead, we used an approach similar to previous work in which we found that greedy sequential learning could be an effective means to identify an optimal combination of groups[4]. To identify the data sources to include in the final model, we combine the two top-ranked data sources into a single kernel and record its balanced accuracy during LOCO-CV. We build subsequent models by adding data sources in descending order of balanced accuracy, constructing a kernel for each combination of data sources. We select as our final combination the data sources associated with the kernel where balanced accuracy reaches a plateau or declines thereafter.

For non-coding regions, the best model incorporated the top five feature groups: *46-way conservation*, *100-way conservation*, *Spectrum*, *Genomic context* and *Mappability* (Figure S6). At 58.0%, the kernel with two feature groups represents an improvement over the best individual kernel (*46-way conservation*, 57.2%, Table S4). Balanced LOCO-CV accuracy continues to increase with additional features until the top five feature groups are combined (60.6% balanced accuracy). From that point onward, additional feature groups provide no evident advantage, as average balanced accuracy declines. Hence our final noncoding-region model uses a single kernel that contains these top five feature groups.

For coding regions, our best model again included the top five data sets: *VEP* (including amino acid substitutions), *46-way conservation*, *100-way conservation*, *Genomic context* and *Spectrum* (Figure S6, right). The first two data sets yield a substantial improvement over either one individually: 69.2% balanced accuracy, compared with 67.4% for the top-ranked VEP kernel alone. Balanced LOCO-CV accuracy continues to increase with additional feature groups until the top five feature groups are combined (over 70% balanced accuracy). Our final coding-region model thus consists of a single kernel containing these top five feature groups.

Alternative classification models

For both coding and noncoding classifiers we investigated a variety of kernel-based models using the scikit-learn package (version 0.17.1)[14]. We selected the package for its relatively robust performance and for the variety of models available. We evaluated seven different classification models to select the one yielding the best performance, and to observe changes in balanced accuracy that could reflect potential overfitting (see Table S5). For each classifier we first used LOCO-CV to establish optimal parameters, then compared the balanced accuracy, averaged over 30 runs, to identify the strongest performers in non-coding and coding regions. For each LOCO fold we used balanced training sets of 2,000 examples. In non-coding regions we found that gradient boosting[15], SVM models (see, e.g.,[12]) and Adaboost[16] yield the highest balanced accuracy, with no significant differences observed between them. In coding regions, gradient boosting and random forests[17] yielded the highest balanced accuracy. Based on these results we selected gradient boosting for our models in both regions.

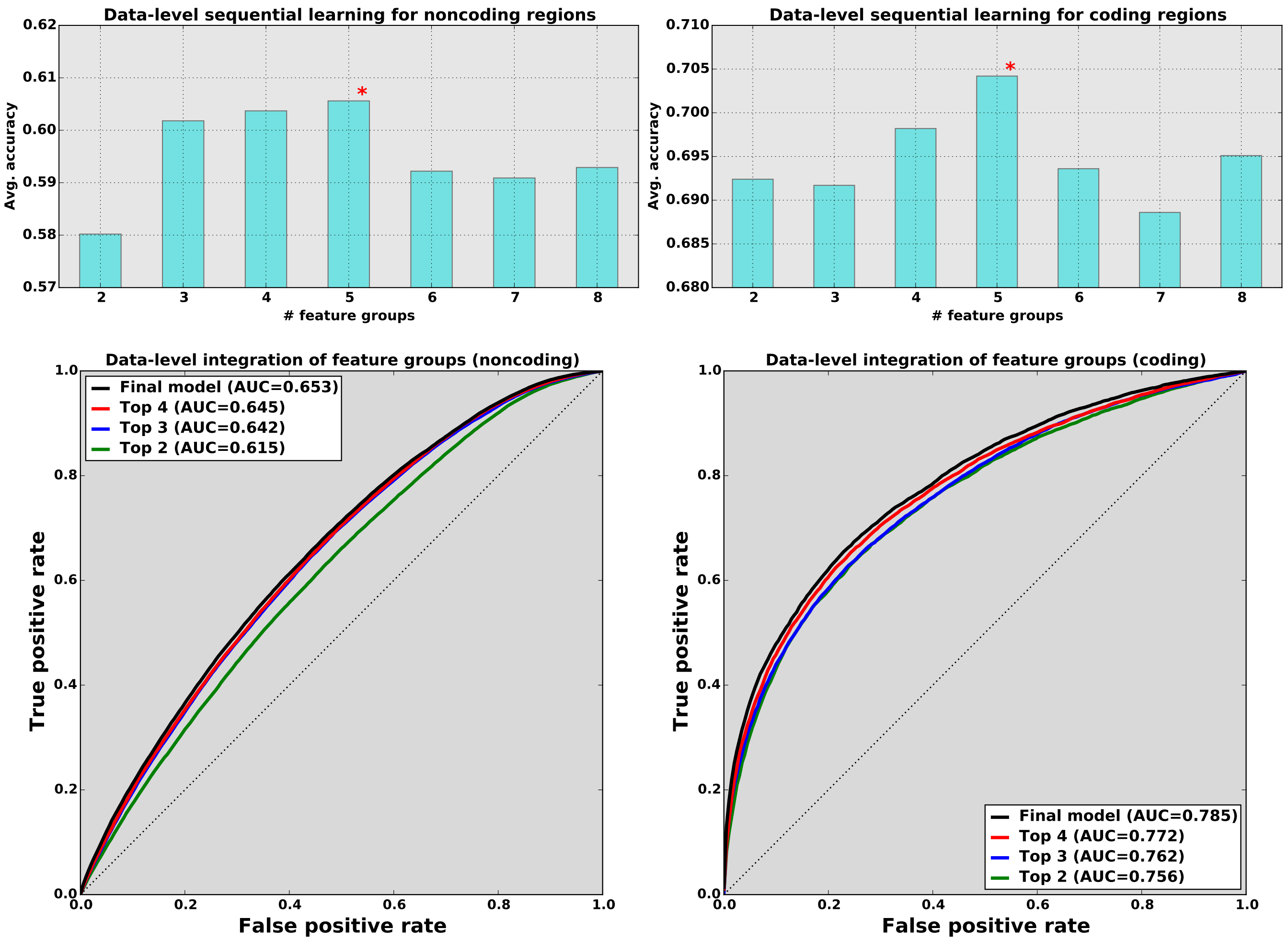

Supplementary Figure S6. Data-level integration for noncoding examples (left) and coding examples For data-level sequential learning we used balanced sets of 2,000 examples in LOCO-CV for each combination of features, and average balanced accuracy over 10 LOCO-CV runs. **Top right:** For non-coding, balanced accuracy peaks at five feature groups (60.6% balanced accuracy) after which it declines slightly. **Top left:** For coding, balanced accuracy also peaks at five feature groups (70.4% balanced accuracy) and declines thereafter. **Bottom row:** ROC curves for the combined kernels constructed during sequential learning reveal the incremental improvement made at each step in the process.

Model	Non-coding	Coding
Gradient boosting	**0.599**	**0.721**
SVM	**0.598**	0.702
Adaboost	**0.594**	0.709
Random forest	0.586	**0.714**
Extra trees	0.581	0.698
Decision tree	0.543	0.635
Naive Bayes	0.527	0.549

Supplementary Table S5. Evaluation of different classification models shows that different models yield the best performance in non-coding and coding regions. Gradient boosting, Adaboost and SVM models all yield comparable balanced accuracy in non-coding regions. In coding regions, Gradient boosting and random forests yield the highest balanced accuracy (values in **bold**). Models were trained in LOCO-CV using balanced training sets of 2,000 examples. Average balanced accuracy was computed over 30 runs. Models are sorted in order of decreasing balanced accuracy on non-coding data.

Kernel-level integration

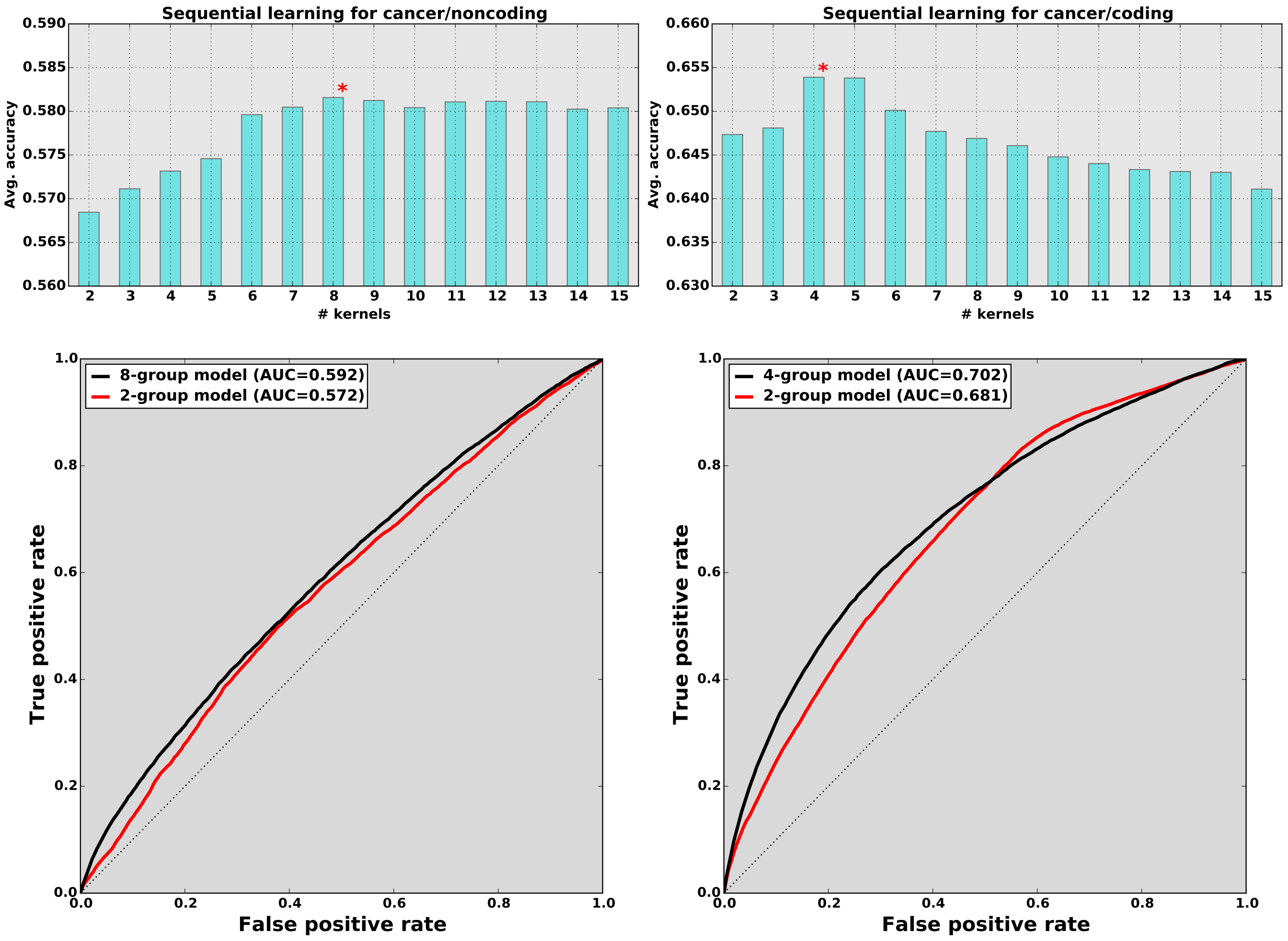

Supplementary Figure S7. MKL aggregate performance on non-coding examples (left) and coding examples (right): (top) sequential learning for aggregate linear models on training data consisting of 1,000 COSMIC ($r \geq 5$) cancer drivers and 1,000 1000-Genomes neutral (presumed non-drivers). Accuracy is averaged over 10 LOCO runs. For non-coding, the simplest two-group model yields 56.8% balanced accuracy in LOCO CV, while balanced accuracy for subsequent aggregate models peaks at eight kernels (58.2% balanced accuracy). For coding regions, the simplest model yields 64.7% balanced accuracy, and subsequent aggregate models achieve a nominal peak at four or five kernels (65.4% balanced accuracy). (bottom) ROC curves for the initial two-group linear model and the top-performing eight-group model illustrate the difference in performance between them.

In previous studies we found that integration at the kernel level, using multiple kernel learning and sequential learning strategies, tended to outperform integration at the data level[3,4,18]. We applied our sequential learning pipeline[4] to examples in both coding and non-coding regions: we ranked the feature groups by balanced accuracy and added them incrementally to aggregate kernels. For non-coding examples, the best of these aggregates achieved just over 58% balanced accuracy (Figure S7). For coding examples, the best achieved over 65% balanced accuracy (Figure S7). However, in both cases, data-level integration yielded at least 4 percentage points higher balanced accuracy than kernel-level integration.

Mis-classified examples

Figure S8 shows distributions of gene features associated with mis-classified examples in coding regions, identified using the Variant Effect Predictor. From these distributions it appears that the coding classifier mis-classifies missense mutations more frequently than other kinds. This phenomenon is most pronounced with the ClinVar test set, however *CScape's* balanced

accuracy on this set was high (91.3%) and hence the number of mis-classified examples is relatively low. By contrast, the differences on the DoCM test set, most carefully curated of the test sets, are less exaggerated. Instead it indicates that the coding classifier mis-classifies some coding mutations that also function as non-coding in some transcripts (*NC transcript* and *Noncoding exon* designations).

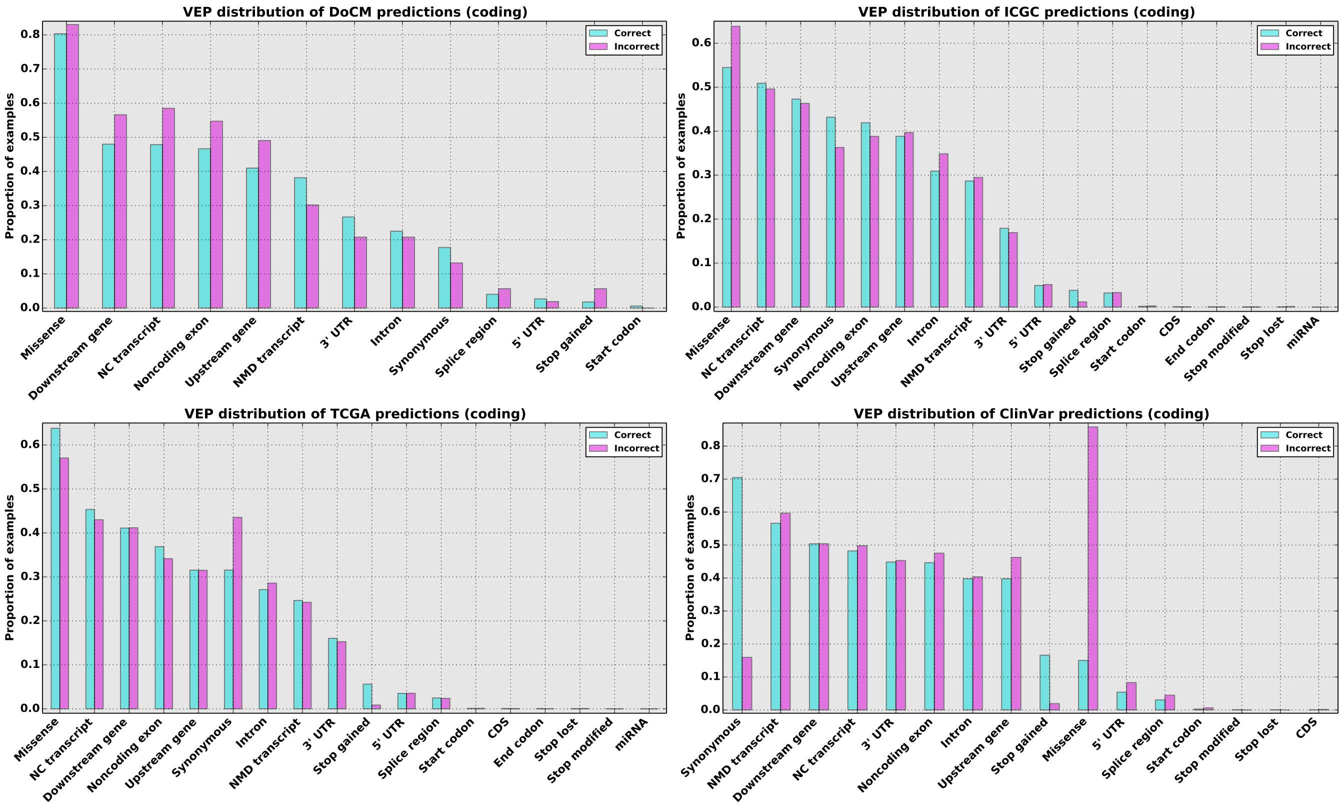

Supplementary Figure S8. Distributions of gene features associated with mis-classified examples in coding regions for DoCM (top left), ICGC (top right), TCGA (bottom left) and ClinVar examples (bottom right) show that the coding classifier tends to mis-classify missense mutations more frequently than other kinds. This is most pronounced in the ClinVar examples (bottom right), however the total number of mis-classified examples is relatively low due to *CScape's* high balanced accuracy on this set. The DoCM test set, the most carefully curated examples, show another trend for the coding classifier to mis-classify coding mutations that also function as non-coding in some transcripts.

When the non-coding model mis-classifies examples, there is no clear preference in comparison with correct classifications (Figure S9). On the relatively small BRCA test set (3,206 examples, compared with 3.3 million for ICGC) the model tends to mis-classify examples designated *NC transcript* and *Noncoding exon* at a slightly higher rate than others.

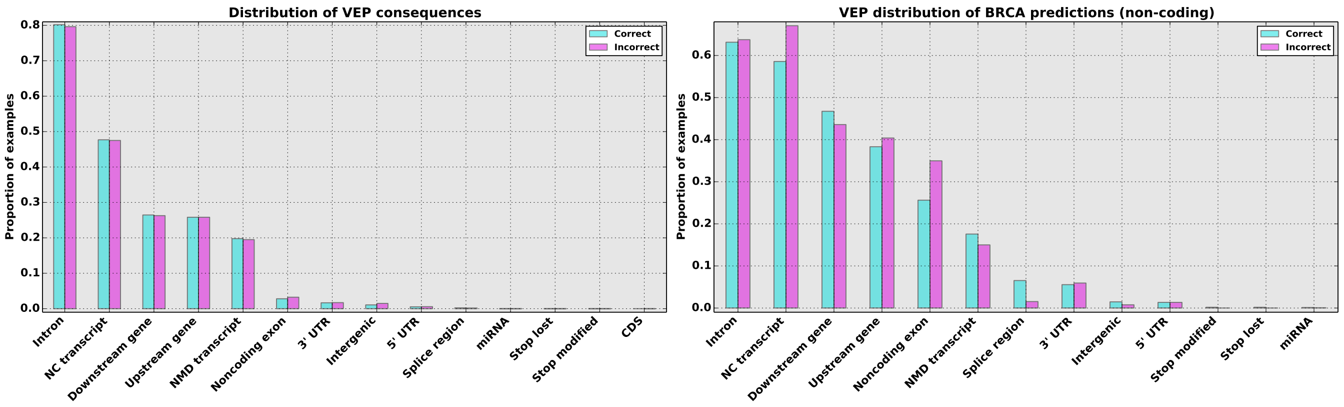

Supplementary Figure S9. Distributions of gene features associated with mis-classified examples in non-coding regions for ICGC (top) and BRCA (TCGA) examples (right) show that the non-coding classifier tends to mis-classify mutations of all kinds at roughly the same frequency.

Regions of interest

To isolate regions of clustered positive predictions, which we call *Regions of Interest* (*RoI*s), we determined the distribution of confidence values and labeled a region as a *RoI* if the contiguous set of positive confidence values located in this region had a very low expectation of being observed, relative to the overall distribution. We denote the p-score confidence associated with the predicted label, at nucleotide position i, by p_i.

The method we used had three parameters labeled c_1, c_2 and w. c_1 is the upper bound on the probability measure coming from the statistical significance test for a potential *RoI*, as described below. We used $c_1 = 10^{-11}$, which is smaller than the reciprocal of the number of nucleotides in the human genome. The parameter c_2 is the lower limit on the magnitude of a positive confidence value for initiation of aggregation of a set of contiguous confidence values for subsequent determination of a possible *RoI*. Thus at the start of accumulation of an *active set*, $p_i > c_2$. At any given nucleotide position there would be three possible variants differing from the reference sequence label, each with a different score for pathogenicity. This means differing patterns of sequence variants can highlight a given *RoI* as pathogenic or neutral. Consequently, we only consider the more basic question of: is variation away from the reference sequence across a given region associated with a statistically significant score. This means we use the average of the three associated scores at a nucleotide position. In addition, as a guide to the value of c_2, we use peak-balanced-test-accuracy confidence thresholds, using such averaged scores, achieved via cautious classification. The determination of these peak balanced accuracy cutoffs follows the narrative for cautious classification given below, except that we are using *averaged* scores. In short, a cutoff of $c_2 = 0.89$, gives a corresponding peak balanced test accuracy of 91.7% (17.7% of coding positions have an average p-score of 0.89 or higher in the test data). For non-coding regions a peak balanced accuracy of 76.1% was achieved at a cutoff $c_2 = 0.70$ (14.8% of test set positions have p-scores greater than this).

After initiation of an active set for investigation, we aggregated confidence values p_i to the active set as follows. Each added value must have a genomic location adjacent to the previous member of the set. If a prediction was absent from a given location, this was sufficient to terminate addition of new values to the active set. We added values if at least one of two criteria was met: $p_i > c_2$ or the value of a moving average exceeded c_2. We used this moving average to prevent noise and variability in the prediction process from breaking up large *RoI*s. The moving average had a flank of size w on each side of the predicted average. We chose $w = 5$, through experimentation and visualisation using our Genome Tolerance Browser[19]. Accumulation of an active set of values for a putative *RoI* therefore ended when the next nucleotide prediction position was no longer adjacent to the current one, or when both p_i and the moving average had fallen below c_2. From the p_i-distribution we can establish a conditional density function (CDF) and then use a Kolmogorov-Smirnov (KS) test to quantify the statistical significance of the set of p_i-scores in the putative *RoI*, taken against the whole distribution generated by the classifier across the genome. The active set was then regarded as a *RoI* if the p-score from the KS test was less than c_1.

Website

For a small number of mutations, one can use the *CScape* website (*cscape.biocompute.org.uk*) to obtain predictions quickly. Mutations are specified using the format `chromosome, position, reference, allele`, with each entry on a separate line. For example, the query for the first result in Figure S10 would be “`1,69094,G,A`”. The results table uses color coding to highlight scores that signify positive or negative predictions, as well as scores that fall above or below cautious classification thresholds. There is also a link to a file that contains the same results in tab-delimited format. For those with a

large number of mutations to classify, the entire database may be downloaded (57GB) along with a script that performs the same function as the website.

Chromosome	Position	Variant	Coding score	Noncoding score
1	69094	G/A	0.346111	
2	230046	T/G	0.950541	
2	962199	G/C		0.570165
5	177113	A/G		0.133258
8	195703	A/G	0.649616	

Supplementary Figure S10. ***CScape* website results page** shows scores for coding and noncoding mutations, color-coded to highlight scores above and below the cautious-classification threshold.

Competing methods

Our proposed models, using different features but a common approach, are designed to predict oncogenic SNVs in both coding and noncoding regions of the genome. Hence we selected distinct sets of state-of-the-art predictors for coding and noncoding regions to compare against our own predictors (see Table S6).

For coding regions, we selected three cancer-specific prediction methods based on the TransFIC approach[20]: MutationAssessor[21], SIFT[22] and PolyPhen-2[10]. These focus on non-synonymous SNVs (nsSNVs) that result in amino acid substitutions, while the other three, CADD[13] DANN[23] and FATHMM-MKL[3], generate predictions for all SNVs. For noncoding regions, we used the general-purpose predictors CADD, DANN and FATHMM-MKL as above, along with FunSeq2[24], the only other cancer-specific predictor we are aware of that was designed for SNVs in noncoding regions. Three of these competitors provide prediction databases for convenience: FATHMM-MKL, CADD and DANN. Hence for these methods we obtained predictions by looking up mutations in each database. For FunSeq2 scores we submitted queries to the associated website and used the noncoding scores from the resulting VCF output files. For TransFIC scores we submitted queries to the associated website and used the TransFIC-adjusted scores for MutationAssessor, PolyPhen2 and SIFT.

Method	mutation types	Cancer-specific	Genomic regions
CScape	all	Yes	all
MutationAssessor (TransFIC)	nsSNV	No	coding
Poly-Phen2 (TransFIC)	nsSNV	No	coding
SIFT (TransFIC)	nsSNV	No	coding
FunSeq2	all	Yes	noncoding
FATHMM-MKL	all	No	all
CADD	all	No	all
DANN	all	No	all

Supplementary Table S6. Methods used in our comparisons. *CScape* is designed to make accurate, cancer-specific predictions for any SNV in any region of the genome. Hence it should be competitive with methods designed specifically for oncogenic mutations (such as TransFIC and FunSeq2) or generic mutations (such as CADD, DANN and FATHMM-MKL), and it should perform as well as methods designed for coding regions, noncoding regions, or both.

Score thresholds

Several of the competing methods publish recommended thresholds for assigning positive (pathogenic) or negative (neutral) labels to test examples: values above the threshold are labeled positive, whilst those below are labeled negative. FATHMM-MKL and DANN both use a threshold of 0.5, while the CADD website recommends a cutoff of 0 on its raw scores. The TransFIC method standardises scores for each underlying method, and divides them into three categories that represent *low impact*, *medium impact* and *high impact* predictions, where the standardised mean 0 falls in the middle range (see[20], Figure 4), hence we use 0 as the default setting for these methods.

FunSeq2 ranks non-coding examples, but no specific cutoff is recommended. Initially we established a cutoff by iterating over all FunSeq2 scores for our non-coding training set and choosing the value that yielded the highest balanced accuracy. However, this procedure yielded a threshold (5.21×10^{-25}) that performed poorly on our test sets, possibly misrepresenting FunSeq2's true potential. Hence we use the same procedure individually for each of the non-coding benchmarks (0.623 on the BRCA set, 0.601 for the ICGC set) and report FunSeq2's peak accuracy on those sets. We then used the closest score (0.56) as the cutoff for known non-coding drivers.

Tables of results

The results in this section have been taken over full test sets, while the figures in the main paper reflect bootstrap statistics that provide confidence intervals.

Statistics definitions

In this work we use several statistics to evaluate classifier performance, including balanced accuracy, sensitivity, specificity, Matthews correlation coefficient (MCC) and positive predictive value (PPV). For any given test set, we count the number of true positives (TP), true negatives (TP), false positives (FP) and false negatives (FN). The statistics are then computed as follows:

$$\begin{aligned}
\text{sensitivity} &= \frac{TP}{TP+FN} \\
\text{specificity} &= \frac{TN}{TN+FP} \\
\text{balanced accuracy} &= 0.5 * (\text{sensitivity + specificity}) \\
\text{PPV} &= \frac{TP}{TP+FP} \\
\text{MCC} &= \frac{TP*TN-FP*FN}{\sqrt{(TP+FP)(TP+FN)(TN+FP)(TN+FN)}}
\end{aligned}$$

When we compare methods, we use bootstrapping to split each test set randomly and we evaluate each method's metrics (above) on the randomised test sets. In most cases we split a test set into 20 subsets, the exception being the DoCM test set that has just 551 positive and 171 negative examples, hence only 10 bootstrap sets were used. We then use a non-parametric, two-sided Mann-Whitney test to compare metrics between methods.

Unseen examples in non-coding regions

Method	Sens.	Spec.	Acc.	MCC	PPV	AUC
CScape (cautious)	**0.79**	0.86	**0.83**	**0.65**	**0.78**	**0.87**
CScape (default)	**0.78**	0.52	**0.65**	**0.31**	**0.62**	**0.71**
CADD	0.53	0.54	0.53	0.07	0.53	0.55
DANN	0.71	0.37	0.54	0.08	0.53	0.55
FunSeq2	0.29	0.74	0.51	0.03	0.53	0.52
FATHMM-MKL	0.07	**0.96**	0.51	0.05	0.60	0.61

Supplementary Table S7. Tests on unseen ICGC examples in non-coding regions show that *CScape* substantially outperforms competitors at both the default threshold and at the cautious classification threshold. The cautious classification threshold for non-coding regions is $\tau = 0.70$, which yields predictions for 19.6% of examples.

Method	Sens.	Spec.	Acc.	MCC	PPV	AUC
CScape (cautious)	**0.99**	0.69	**0.84**	**0.55**	**0.45**	**0.90**
CScape (default)	**0.95**	0.46	**0.70**	0.26	0.18	**0.84**
FATHMM-MKL	0.55	**0.86**	**0.70**	**0.33**	**0.33**	0.80
CADD	0.81	0.48	0.65	0.19	0.17	0.75
FunSeq2	0.53	0.73	0.63	0.18	0.20	0.68
DANN	0.88	0.37	0.62	0.17	0.15	0.75

Supplementary Table S8. Tests on unseen BRCA (TCGA) examples in non-coding regions show that *CScape* yields state-of-the art balanced accuracy and AUC scores at the default threshold. At the cautious classification threshold ($\tau = 0.70$), CScape yields higher scores than competitors in all categories except specificity, whilst producing predictions for 24.8% of examples.

Unseen examples in coding regions

Method	Sens.	Spec.	Acc.	MCC	PPV	AUC
CScape (cautious)	**1.00**	0.50	0.75	0.70	**0.99**	0.73
CScape (default)	0.97	**0.79**	**0.89**	**0.79**	0.94	**0.96**
FATHMM-MKL	0.97	0.69	0.83	0.71	0.91	0.94
DANN	**0.99**	0.17	0.58	0.34	0.79	0.94
CADD	**0.99**	0.24	0.62	0.41	0.81	0.95
MutationAssessor	0.75	0.43	0.59	0.11	0.94	0.69
PolyPhen2	0.89	0.66	0.77	0.42	**0.97**	0.88
SIFT	0.91	0.50	0.70	0.33	0.95	0.81

Supplementary Table S9. Tests on unseen DoCM examples in coding regions show that *CScape* yields higher balanced accuracy than other methods when all methods are evaluated using default thresholds. At the cautious classification threshold *CScape* makes predictions for 275 out of 724 examples. Just eight of these are negatives, a severe imbalance that leads to relatively low balanced accuracy, but sensitivity is 100% and the PPV is 99%, providing evidence that *p*-scores above the cautious threshold correctly identify oncogenic examples. (MCC=Matthews correlation coefficient, PPV=positive predictive value, AUC=area under ROC curve)

Method	Sens.	Spec.	Acc.	MCC	PPV	AUC
CScape (cautious)	**1.00**	**0.85**	**0.93**	**0.89**	**0.93**	0.96
CScape (default)	0.95	**0.87**	**0.91**	**0.73**	**0.66**	**0.97**
PolyPhen2	0.85	0.55	0.70	0.36	0.41	0.79
SIFT	0.90	0.46	0.68	0.33	0.39	0.80
FATHMM-MKL	0.73	0.56	0.64	0.23	0.30	0.76
MutationAssessor	0.92	0.37	0.64	0.28	0.34	0.80
CADD	**0.99**	0.27	0.63	0.26	0.27	**0.97**
DANN	**0.99**	0.20	0.60	0.22	0.25	0.93

Supplementary Table S10. Tests on unseen ClinVar examples in coding regions show that *CScape* at the default threshold yields balanced accuracy substantially higher than competitors. At the cautious classification threshold, CScape yields exceptionally high statistics in all categories. (MCC=Matthews correlation coefficient, PPV=positive predictive value, AUC=area under ROC curve)

Method	Sens.	Spec.	Acc.	MCC	PPV	AUC
CScape (cautious)	**0.94**	**0.80**	**0.87**	**0.72**	**0.95**	**0.88**
CScape (default)	0.61	**0.78**	**0.70**	**0.39**	0.81	0.75
PolyPhen2	0.63	0.70	0.66	0.29	**0.84**	0.72
FATHMM-MKL	0.76	0.53	0.65	0.30	0.70	0.73
MutationAssessor	0.74	0.54	0.64	0.27	0.79	0.70
SIFT	0.68	0.59	0.64	0.25	0.82	0.68
CADD	0.91	0.34	0.62	0.31	0.67	**0.78**
DANN	**0.93**	0.24	0.59	0.25	0.64	0.76

Supplementary Table S11. Tests on unseen TCGA examples in coding regions show that *CScape* yields higher balanced accuracy than other methods when all methods are evaluated using default thresholds. In addition, *CScape's* PPV and AUC scores are competitive with the best alternatives. CADD and DANN, modeled from the same training set, yield the highest sensitivity but the lowest specificity. At the peak threshold $\tau = 0.89$ established for cautious classification, CScape makes confident predictions for 17,801 test examples (16.3%) yielding the highest performance statistics in all categories. (MCC=Matthews correlation coefficient, PPV=positive predictive value, AUC=area under ROC curve)

Method	Sens.	Spec.	Acc.	MCC	PPV	AUC
CScape (cautious)	0.91	0.66	**0.78**	**0.59**	**0.87**	**0.81**
CScape (default)	0.59	0.72	0.65	0.31	0.63	0.71
FATHMM-MKL	0.64	**0.73**	**0.68**	**0.37**	0.66	**0.74**
PolyPhen2	0.60	0.70	0.65	0.29	**0.74**	0.70
MutationAssessor	0.73	0.53	0.63	0.27	0.69	0.69
SIFT	0.68	0.59	0.63	0.26	0.71	0.68
CADD	0.89	0.30	0.60	0.24	0.52	**0.74**
DANN	**0.93**	0.22	0.58	0.21	0.50	**0.74**

Supplementary Table S12. Tests on unseen ICGC examples in coding regions show that *CScape* at the default threshold yields balanced accuracy slightly lower than its top competitor, FATHMM-MKL, while PolyPhen2 yields by far the highest PPV. At the cautious classification threshold, CScape yields substantially higher balanced accuracy, MCC, PPV and AUC scores than all other methods, producing predictions for 13.1% of examples. (MCC=Matthews correlation coefficient, PPV=positive predictive value, AUC=area under ROC curve)

Cancer-specific thresholds

	Non-coding		Coding			
Method	ICGC	BRCA	ClinVar	DoCM	ICGC	TCGA
CScape (default)	**0.65**	**0.70**	**0.91**	**0.89**	0.65	**0.70**
FATHMM-MKL	0.51	**0.70**	0.64	0.83	**0.68**	**0.70**
($t = 0.08$)	0.58	0.63	0.60	0.69	0.64	0.65
CADD	0.53	0.64	0.63	0.62	0.60	0.62
($t = 0.15$)	0.54	0.67	0.65	0.63	0.61	0.64
DANN	0.54	0.63	0.60	0.58	0.58	0.59
($t = 0.54$)	0.54	0.64	0.62	0.61	0.59	0.60

Supplementary Table S13. Thresholds optimised on the *CScape* training data yield mixed results for competing methods. By assessing balanced accuracy for competing methods over the full range of their scores on the training data, we may establish alternative cutoffs (t) for discriminating between oncogenic and neutral predictions. For each method, we compare the balanced accuracy depicted in Figures 4 and 5 in the main paper with balanced accuracy using these new thresholds. For non-coding benchmarks, CADD and DANN both improve slightly when we apply these optimised thresholds, while FATHMM-MKL has mixed results. For coding benchmarks, CADD and DANN improve across all tests, while FATHMM-MKL suffers worse performance, with balanced accuracy dropping by as many as 14 percentage points. The coding-region predictors all exhibit better performance on the ClinVar and DoCM benchmarks, but generally show reduced performance on ICGC and TCGA.

Many of the methods we assess have been designed or refined for oncogenic variants, but the three genome-wide methods, CADD, DANN and FATHMM-MKL were developed as general-purpose predictors for germline variants. For these, we wanted to explore the possibility of enhancing performance by establishing optimal cancer-specific thresholds. Hence we established a threshold for each method by iterating over all its scores on our combined (coding plus non-coding) training data, then choosing as its cancer-specific threshold the score that yielded the highest balanced accuracy. This yielded cancer-specific thresholds of 0.15 for CADD, 0.54 for DANN, and a low value of 0.08 for FATHMM-MKL. We then applied these thresholds and re-assessed each method on our benchmark test sets (Supplementary Table S13). CADD and DANN both improved slightly (up to 3 percentage points) on tests in both coding and non-coding regions when we applied these optimised thresholds. FATHMM-MKL exhibited mixed results: balanced accuracy improved by 7 percentage points on the ICGC test, but declined on the remaining tests, with a full 14 percentage point drop on the DoCM benchmark.

Region-specific thresholds

	Non-coding			Coding				
Method	t	ICGC	BRCA	t	ClinVar	DoCM	ICGC	TCGA
CScape (default)	—	**0.65**	**0.70**	—	0.91	0.89	0.65	**0.70**
CScape (cautious)	—	**0.83**	**0.84**	—	**0.93**	0.75	**0.78**	**0.87**
FATHMM-MKL	—	0.51	**0.70**	—	0.64	0.83	**0.68**	**0.70**
(optimised)	0.08	0.58	0.63	0.78	0.70	0.87	**0.68**	0.69
CADD	—	0.53	0.64	—	0.63	0.62	0.60	0.62
(optimised)	0.15	0.54	0.67	2.67	**0.93**	**0.91**	**0.68**	**0.71**
DANN	—	0.54	0.63	—	0.60	0.58	0.58	0.59
(optimised)	0.54	0.54	0.64	0.96	0.91	0.89	**0.69**	**0.71**

Supplementary Table S14. Region-specific thresholds optimised on the *CScape* training data yield substantial improvements for CADD and DANN in coding regions. By optimising thresholds for non-coding and coding regions, we were able to boost CADD's and DANN's performance on the coding region benchmarks.

Taking this idea a step further, we investigated using thresholds specific to each region (coding or non-coding). For all three methods, the non-coding region threshold was nearly the same as the genome-wide thresholds, so performance in non-coding regions was the same (Table S14). This is not too surprising, as the non-coding training set contains nearly three times as many examples as the coding set and thus would have greatly influenced the genome-wide thresholds. All three methods benefitted from coding-region thresholds substantially higher than their defaults: FATHMM-MKL's optimal coding threshold was $t = 0.78$, compared with a default threshold of 0.5; CADD's optimal threshold was 2.67 compared with a default of 0, while DANN's optimal threshold of 0.96 was close to the top of its range of $[0, 1]$. CADD and DANN both improved markedly on the coding benchmark tests, achieving the highest accuracy of all methods on the DoCM coding benchmark, with accuracy of 91% and 90%, respectively. CADD achieved similar performance on the ClinVar benchmark, at 93%. Hence, using thresholds optimised on a cancer-specific dataset, it may be possible for general-purpose methods to perform as well as *CScape* in coding regions, and in some cases, slightly better. However, we believe this kind of procedure would be burdensome for most users, and note that *CScape* achieves similar or higher accuracy even at its default threshold.

ROC curves

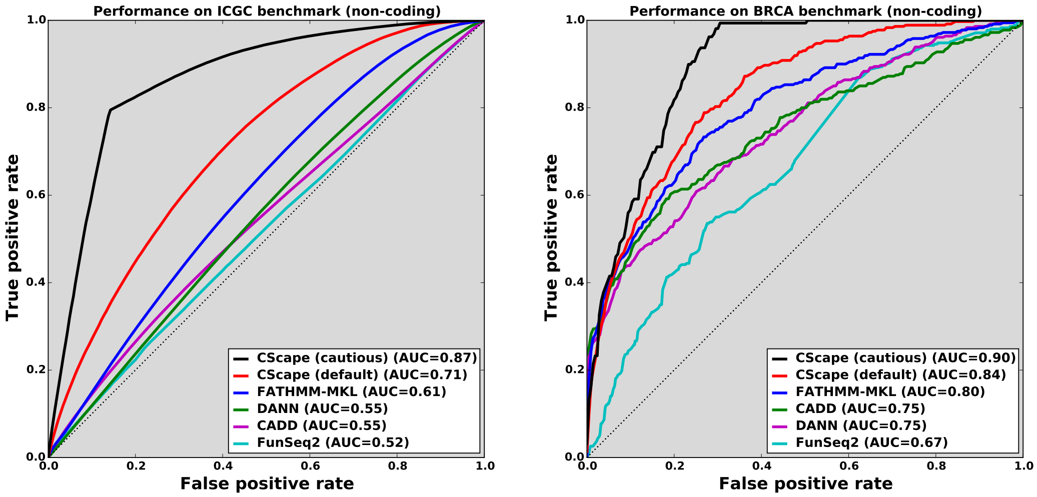

Supplementary Figure S11. Comparison of ROC curves for all methods in non-coding regions illustrates the differences between them. Cautious classification creates a discontinuity in the rankings for *CScape (cautious)*, which appears as a kink in the curve (black line). In both benchmark tests, *CScape (default)* yields stronger performance than competing methods, while cautious classification *CScape (cautious)* pushes performance higher still.

The results above show that when we use cautious classification, performance measures are likely improve, often significantly. To gain a better understanding of exactly how cautious classification behaves, we also compare ROC curves for *CScape* with those of the other methods tested. For non-coding predictions, cautious classification only accepts positive predictions with $p \geq 0.70$ and negative predictions with $p \leq 0.30$. This creates a discontinuity as there are no values between 0.30 and 0.70 in the rankings, and appears as a kink in the ROC curves for cautious classification (black lines in both ICGC and DoCM plots, Supplementary Figure S11).

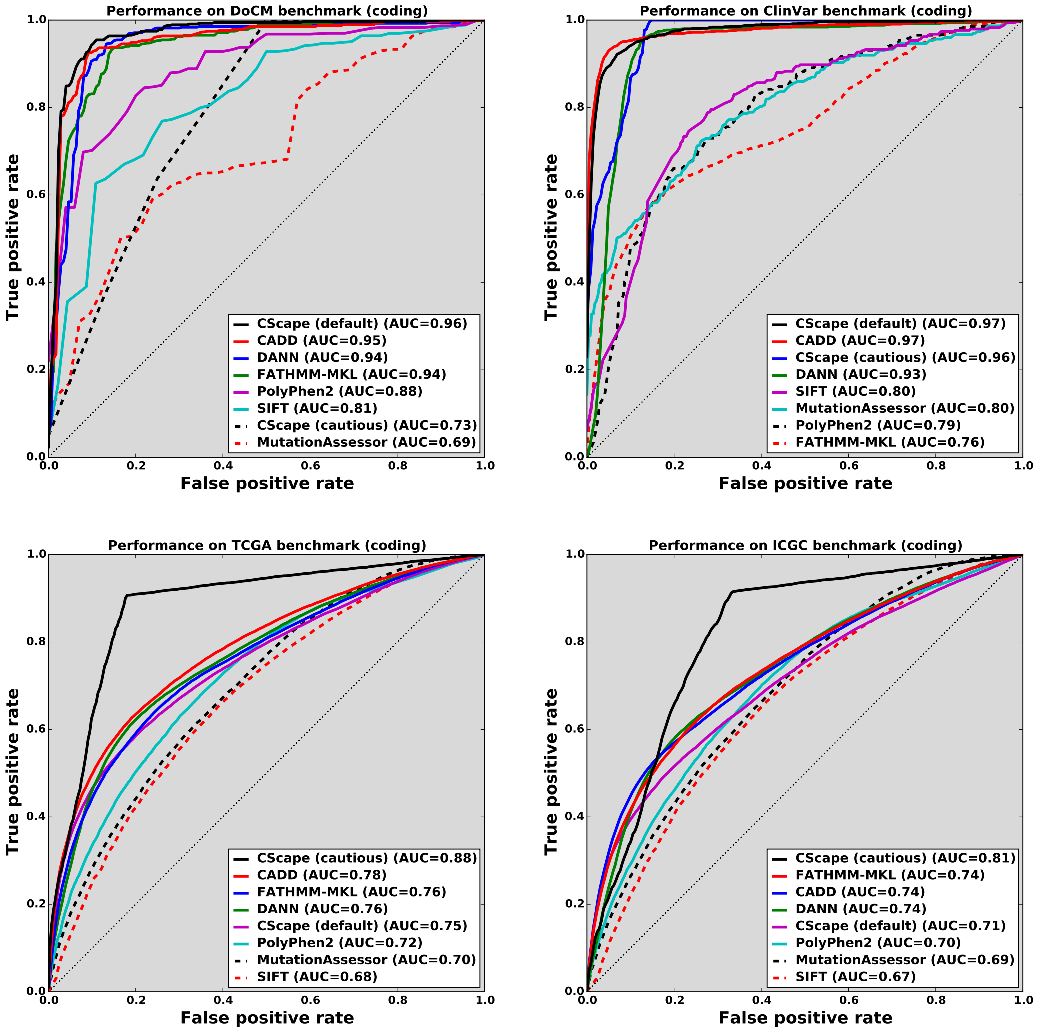

Supplementary Figure S12. Comparison of ROC curves for all methods in non-coding regions illustrates the differences between them. In the DoCM and ClinVar benchmarks (top row) DANN, CADD and *CScape (default)* all yield strong performance with AUC scores above 0.90. Cautious classification performs relatively poorly on DoCM, but matches or outperforms the other methods on the remaining sets. Again, we see a noticeable kink in the *CScape (cautious)* curve with its discontinuous ranking set.

For coding predictions, cautious classification only accepts positive predictions with $p \geq 0.89$ and negative predictions with $p \leq 0.11$. This creates an exaggerated discontinuity, as no values between 0.11 and 0.89 appear in the rankings. Many of the correctly predicted DoCM examples fall within this range, so the *CScape (cautious)* rankings are not as reliable as for the remaining benchmark sets (Supplementary Figure S12). DANN, CADD and *CScape (default)* all perform well on the DoCM and ClinVar benchmarks, while none of the methods performs especially well on the TCGA and ICGC benchmarks, whilst *CScape (cautious)* provides better rankings.

References

1. Forbes, S. A. *et al.* Cosmic: mining complete cancer genomes in the catalogue of somatic mutations in cancer. *Nucleic acids research* **39**, D945–D950 (2010).
2. The 1000 Genomes Project Consortium. An integrated map of genetic variation from 1,092 human genomes. *Nat.* **491**, 56–65 (2012).
3. Shihab, H. *et al.* An integrative approach to predicting the functional effects of non-coding and coding sequence variation. *Bioinforma.* **31**, 1536–1543 (2015).
4. Rogers, M. *et al.* Sequential data selection for predicting the pathogenic effects of sequence variation. In *Bioinformatics and Biomedicine (BIBM), 2015 IEEE International Conference on*, 639–644 (2015).
5. Pollard, K. S., Hubisz, M., Rosenbloom, K. & Siepel, A. Detection of non-neutral substitution rates on mammalian phylogenies. *Genome Res.* **20**, 110–121 (2010).
6. Siepel, A. *et al.* Evolutionarily conserved elements in vertebrate, insect, worm, and yeast genomes. *Genome Res.* **15**, 1034–1050 (2005).
7. Blanchette, M. *et al.* Aligning multiple genomic sequences with the threaded blockset aligner. *Genome research* **14**, 708–715 (2004).
8. Consortium, T. E. P. An integrated encyclopedia of DNA elements in the human genome. *Nat.* **489**, 57–74 (2012).
9. Kundaje, A. *et al.* Integrative analysis of 111 reference human epigenomes. *Nat.* **518**, 317–330 (2015).
10. Adzhubei, I. *et al.* A method and server for predicting damaging missense mutations. *Nat. Methods* **7**, 248–249 (2010).
11. Leslie, C. S., Eskin, E. & Noble, W. S. The spectrum kernel: A string kernel for SVM protein classification. In *Pacific Symposium on Biocomputing*, vol. 7, 566–575 (World Scientific, 2002).
12. Campbell, C. & Ying, Y. *Learning with Support Vector Machines* (Morgan and Claypool, 2011).
13. Kircher, L.A. *et al.* A general framework for estimating the relative pathogenicity of human genetic variants. *Nat. Genet.* **46**, 310–315 (2014).
14. Pedregosa, F. *et al.* Scikit-learn: Machine learning in Python. *J. Mach. Learn. Res.* **12**, 2825–2830 (2011).
15. Schapire, R. E. The boosting approach to machine learning: An overview. In *Nonlinear estimation and classification*, 149–171 (Springer, 2003).
16. Freund, Y. & Schapire, R. E. A decision-theoretic generalization of on-line learning and an application to boosting. In *European conference on computational learning theory*, 23–37 (Springer, 1995).
17. Ho, T. K. Random decision forests. In *Document Analysis and Recognition, 1995., Proceedings of the Third International Conference on*, vol. 1, 278–282 (IEEE, 1995).
18. Shihab, H., Rogers, M., Gaunt, T. & Campbell, C. HIPred: an integrative approach for predicting haploinsufficiency in the human genome. *press* (2017).
19. Shihab, H. A., Rogers, M. F., Ferlaino, M., Campbell, C. & Gaunt, T. R. GTB–an online genome tolerance browser. *BMC Bioinformatics* **18**, 20 (2017).
20. Gonzalez-Perez, A., Deu-Pons, J. & Lopez-Bigas, N. Improving the prediction of the functional impact of cancer mutations by baseline tolerance transformation. *Genome medicine* **4**, 1 (2012).
21. Reva, B., Antipin, Y. & Sander, C. Predicting the functional impact of protein mutations: application to cancer genomics. *Nucleic Acids Res.* **39**, e118 (2011).
22. Kumar, P., Henikoff, S. & Ng, P. Predicting the effects of coding non-synonymous variants on protein function using the SIFT algorithm. *Nat. Protoc.* **4**, 1073–81 (2009).
23. Quang, D., Chen, Y. & Xie, X. DANN: a deep learning approach for annotating the pathogenicity of genetic variants. *Bioinforma.* **31**, 761–763 (2014).
24. FunSeq2: a framework for prioritizing noncoding regulatory variants in cancer. *Genome Biol.* **15** (2014).
